# Next-Generation Sequencing–Based Testing Among Patients With Advanced or Metastatic Nonsquamous Non–Small Cell Lung Cancer in the United States: Predictive Modeling Using Machine Learning Methods

**DOI:** 10.2196/64399

**Published:** 2025-06-11

**Authors:** Alan James Michael Brnabic, Ilya Lipkovich, Zbigniew Kadziola, Dan He, Peter M Krein, Lisa M Hess

**Affiliations:** 1 Eli Lilly and Company Sydney Australia; 2 Eli Lilly and Company Indianapolis, IN United States; 3 Syneos Health Morrisville, NC United States

**Keywords:** lung cancer, NGS testing, next-generation sequencing, real-world data, machine learning, biomarkers, predictive modeling, artificial intelligence, treatment guidelines, tumor biomarker, oncology

## Abstract

**Background:**

Next-generation sequencing (NGS) has become a cornerstone of treatment for lung cancer and is recommended in current treatment guidelines for patients with advanced or metastatic disease.

**Objective:**

This study was designed to use machine learning methods to determine demographic and clinical characteristics of patients with advanced or metastatic non–small cell lung cancer (NSCLC) that may predict likelihood of receiving NGS-based testing (ever vs never NGS-tested) as well as likelihood of timing of testing (early vs late NGS-tested).

**Methods:**

Deidentified patient-level data were analyzed in this study from a real-world cohort of patients with advanced or metastatic NSCLC in the United States. Patients with nonsquamous disease, who received systemic therapy for NSCLC, and had at least 3 months of follow-up data for analysis were included in this study. Three strategies, logistic regression models, penalized logistic regression using least absolute shrinkage and selection operator penalty, and extreme gradient boosting with classification trees as base learners, were used to identify predictors of ever versus never and early versus late NGS testing. Data were split into D1 (training+validation; 80%) and D2 (testing; 20%) sets; the 3 strategies were evaluated by comparing their performance on multiple *m*=1000 splits in the training (70%) and validation data (30%) within the D1 set. The final model was selected by evaluating performance using the area under the receiver operating curve while taking into account considerations of simplicity and clinical interpretability. Performance was re-estimated using the test data D2.

**Results:**

A total of 13,425 met the criteria for the ever NGS-tested, and 17,982 were included in the never NGS-tested group. Performance metrics showed the area under the receiver operating curve evaluated from validation data was similar across all models (77%-84%). Among those in the ever NGS-tested group, 84.08% (n=11,289) were early NGS-tested, and 15.91% (n=2136) late NGS-tested. Factors associated with both ever having NGS testing as well as early NGS testing included later year of NSCLC diagnosis, no smoking history, and evidence of programmed death ligand 1 testing (all *P*<.05). Factors associated with a greater chance of never receiving NGS testing included older age, lower performance status, Black race, higher number of single-gene tests, public insurance, and treatment in a geography with Molecular Diagnostics Services Program adoption (all *P*<.05).

**Conclusions:**

Predictors of ever versus never as well as early versus late NGS testing in the setting of advanced or metastatic NSCLC were consistent across machine learning methods in this study, demonstrating the ability of these models to identify factors that may predict NGS-based testing. There is a need to ensure that patients regardless of age, race, insurance status, and geography (factors associated with lower odds of receiving NGS testing in this study) are provided with equitable access to NGS-based testing.

## Introduction

The care of patients with non–small cell lung cancer (NSCLC) has changed dramatically since the early 2010s, from a chemotherapy-based approach that was tailored only to the disease histology (squamous or nonsquamous tumors) to becoming a disease with multiple actionable biomarkers that can identify targeted therapies associated with superior outcomes based on individual patient genomic characteristics [1,2]. This has led to the adoption of next-generation sequencing (NGS) recommendations included in treatment guidelines for patients with NSCLC [3].

Unfortunately, despite these recommendations, multiple studies have shown that NGS-based testing is not being used for all patients with advanced or metastatic NSCLC, and only about half of all patients in some studies receive comprehensive biomarker testing [4-6]. The reasons for the lack of testing are unclear but may include barriers to ordering tests, insufficient tissue, clinical deterioration, or a crisis that requires immediate care [6]. More recent studies have also demonstrated a racial disparity in receipt of biomarker testing; patients who are Black are significantly less likely than those who are White to receive NGS-based testing in the United States [7].

Studies evaluating the barriers to testing have typically taken a specific hypothesis-driven a priori categorization of potential barriers to investigate the lack of testing [6,7]. While certainly this approach is critical to investigate specific issues such as racial disparities, this falls short when trying to evaluate the complexity of care and the multiple and potentially interacting factors. Clinical prediction models are an alternative approach to using patient-level evidence to help inform health care decision makers about patient care. These models have been used for decades by health care professionals [8]. Traditionally, prediction models combine patient demographic, clinical, and treatment characteristics in the form of a statistical or mathematical model, usually regression, classification, or neural networks, but deal with a limited number of predictor variables (usually below 25). Flexible machine learning methods can be used, by which the researcher does not force the model to evaluate a limited set of covariates, but rather the models themselves learn by trial and error from the data to make predictions, without having a predefined set of rules for decision-making. Simply, machine learning can be better understood as “learning from data” [9]. The setting of biomarker testing provides an opportunity to apply these methods to more thoroughly explore the factors that are associated with the lack of recommended biomarker testing.

While machine learning methods have been more commonly used for biomarker identification and treatment selection, there is little evidence of these methods applied to the prediction of biomarker testing itself. To date, the investigations surrounding the gaps in biomarker testing have remained largely limited to descriptive research and opinion pieces [10-13]. Therefore, this study was designed to fill this gap in evidence by applying machine learning methods to the question of biomarker testing for patients with advanced or metastatic nonsquamous NSCLC to determine demographic and clinical characteristics that may predict receipt of NGS-based testing. A second objective was to further determine the characteristics that predict receipt of NGS-based testing (early testing) in accordance with clinical guidelines that can inform first-line therapy (vs those who receive NGS-based testing after the first-line therapy is underway). These objectives were pursued to better understand factors associated with experiencing barriers to recommended testing and the timing of such testing to inform future intervention strategies.

## Methods

### Data Source

This study used the Advanced NSCLC Analytic Cohort from the nationwide Flatiron Health electronic health record–derived longitudinal database, comprising deidentified patient-level structured and unstructured data, curated via technology-enabled abstraction [14,15]. The data are deidentified and subject to obligations to prevent reidentification and protect patient confidentiality and are not considered human participants in accordance with the US Code of Federal Regulations [16]. These deidentified data originate from approximately 280 cancer clinics (~800 sites of care) in the United States. Patients in this database are those who have lung cancer *ICD* (*International Classification of Diseases*) codes 162.x (*ICD-9* [*International Classification of Diseases, Ninth Revision*]), C34x, or C39.9 (*ICD-10* [*International Statistical Classification of Diseases, Tenth Revision*]) on at least 2 documented clinical visits on different days occurring on or after January 1, 2011. Longitudinal patient-level data were available through November 2021. Patients must further have had pathology consistent with NSCLC and have advanced or metastatic disease (diagnosed with stage IIIB, IIIC, IVA, or IVB disease or diagnosed with early-stage NSCLC and subsequently developed recurrent or progressive disease).

### Definitions of NGS Testing Cohorts

Patients were included in this analysis if they were in the Flatiron Health Advanced NSCLC Analytic Cohort, had nonsquamous NSCLC, evidence of receipt of systemic therapy, and at least 3 months of follow-up in the database. Receipt of testing by NGS is a field recorded in the electronic medical record database by the health care provider that was used for testing identification in this study. The method of NGS testing (tissue or circulating tumor) is not specified. Patients were excluded who had evidence of NGS-based testing more than 20 days prior to initial NSCLC diagnosis. Patients meeting the inclusion criteria for this study were categorized into 2 groups. The ever NGS-tested group included patients with at least 1 NGS test recorded in the database. All remaining patients were included in the never NGS-tested group, as this group was comprised of patients with no evidence of any NGS test recorded in the database. Among those in the ever NGS-tested group, individuals were further subgrouped by the timing of NGS-based testing. Each patient in the ever NGS-tested group was either included in the early NGS-tested subgroup, including patients whose first or only NGS-based test occurred prior to the start of first-line therapy through day 7 of first-line therapy, or the late NGS-tested subgroup, all remaining patients whose first NGS-based test occurred 8 days or later after the start of first-line therapy. The date of advanced or metastatic diagnosis was considered the index diagnosis date.

### Candidate Predictors

Candidate predictors for receipt and timing of NGS-based testing were prespecified based on published literature, analyses of real-world data, and expert input from the field of cancer diagnostics [4,7,17]. These variables included patient age at advanced or metastatic diagnosis date (years), sex (male or female), race (Asian, Black, White, and other), insurance type (public, private, or other), Eastern Cooperative Oncology Group (ECOG) performance status (0-4), smoking history (ever vs never smoker), body weight (kilograms), BMI (kg/m^2^), practice setting (academic or community), practice volume (the average number of those with NSCLC receiving care at the site where the included patient received care by index year over the period 2011 to 2021), biomarker result (positive, not positive, and not tested) by each available biomarker (anaplastic lymphoma kinase [ALK]; epidermal growth factor receptor [EGFR]; V-Raf murine sarcoma viral oncogene homolog B [BRAF]; Kirsten rat sarcoma virus [KRAS]; c-ros oncogene 1 [ROS1]; mesenchymal epithelial transition [MET]; neurotrophic tyrosine receptor kinase [NTRK]; rearranged during transfection [RET]; and programmed death ligand 1 [PD-L1]), stage of disease at initial diagnosis (0-IV), laboratory value (low, normal, high, or not tested) by blood test (alkaline phosphatase, alanine transaminase, aspartate transferase, bilirubin, creatinine, lymphocyte count, red blood cell count, hematocrit, platelet count, white blood cell count, and hemoglobin), number of non-NGS biomarker tests received (total number of fluorescence in situ hybridization, immunohistochemistry, polymerase chain reaction, or other non-NGS–based tests), as well as 2 variables to identify periods of environmental changes. The first of these variables categorized the status of National Comprehensive Cancer Network (NCCN) Clinical Guidelines: prior to 2016, before NGS was recommended in the guidelines; 2016-2019, when broad-based testing was recommended; and 2020 and later, when NGS-based testing was recommended [18]. The second variable evaluated the timing of US Food and Drug Administration approval of drugs that targeted the available biomarkers: period (1) January 1, 2011-August 25, 2011 (EGFR drugs only); period (2) August 26, 2011-March 10, 2016 (EGFR+ALK); period (3) March 11, 2016-June 21, 2017 (EGFR+ALK+ROS1); period (4) June 22, 2017-November 25, 2018 (EGFR+ALK+ROS1+BRAF); period (5) November 26, 2018-May 5, 2020 (EGFR+ALK+ROS1+BRAF+NTRK); period (6) May 6, 2020-May 26, 2021 (EGFR+ALK+ROS1+BRAF+NTRK+MET+RET); and period (7) May 27, 2021, and later (EGFR+ALK+ROS1+BRAF+MET+NTRK+RET+KRAS) [19]. Additionally, candidate predictors of Medicare Administrative Contractor (MAC) region [20] and Molecular Diagnostics Services (MolDX) Program adoption (yes or no) [21] were included. These variables explored the policies in place at the geography in which the patient received care. MACs are private companies that process claims for Medicare beneficiaries. These companies are geographically distinct and identifiable by unique alphanumeric designations (eg, J8=jurisdiction 8) and by private company names (eg, Noridian and Palmetto) [22]. The MolDX Program determines the coverage of diagnostic testing in 4 MACs across 28 states [20,21]. Importantly, all candidate predictor variables were required to be recorded prior to the end of the early NGS testing period to ensure that no covariates were recorded after the measurement of the NGS testing outcome.

The following interactions were deemed to be clinically relevant and forced into the models for evaluation: smoking and sex, smoking and NCCN guideline periods, race and insurance type, age and ECOG performance status, MAC region and public insurance, and MolDX region and public insurance. The estimates of the expected direction of these relationships were defined in the study protocol and are summarized in Table 1.

**Table 1:** Expected direction of candidate predictors for next-generation sequencing (NGS) testing.

Candidate predictor variable	Expected direction
Year of advanced or metastatic diagnosis	As year increases, NGS testing is more likely.
Smoking status (yes vs no)	Smoking=no, NGS testing is more likely.
Sex (male vs female)	Sex=female, NGS testing is more likely.
Race (Asian, Black, White, other)	Race=Asian or White, NGS testing is more likely.
Practice volume (continuous)	As practice volume increases, NGS testing is more likely.
BMI (using WHO^a^ categories)	BMI=underweight, NGS testing is less likely.
ECOG^b^ performance status (0, 1, 2, 3, or 4)	As ECOG performance status increases, NGS testing is less likely.
Body weight (continuous, in kilograms)	As weight increases, NGS testing is more likely.
Stage at initial diagnosis (0-I, II, III, or IV)	Stage 0-II=NGS is more likely than stage III; stage IV=NGS is more likely than stage III.
EGFR^c^ (not tested, positive, not positive) by non-NGS test	EGFR=positive, NGS less likely.
ROS1^d^ (not tested, positive, not positive) by non-NGS test	ROS1=positive, NGS less likely.
ALK^e^ (not tested, positive, not positive) by non-NGS test	ALK=positive, NGS less likely.
BRAF^f^ (not tested, positive, not positive) by non-NGS test	BRAF=positive, NGS less likely.
KRAS^g^ (not tested, positive, not positive) by non-NGS test	KRAS=positive, NGS less likely.
PD-L1^h^ (not tested, positive, not positive)	PD-L1=positive, NGS less likely.
Number of single-gene tests (continuous)	As the number of single-gene tests increase, NGS less likely.
Practice setting (academic, community)	Practice setting=academic, NGS more likely.
Insurance status (public, private, other)	This relationship is unknown. It is possible that insurance status=public, NGS less likely; however, it is possible that in some cases, insurance status=private only, NGS could be less likely.
MAC^i^ region	No direction is known.
MolDX^j^	While this only applies to Medicare, states may adopt broader policies, and the relationship is uncertain. MolDX may make NGS more likely, but it is largely unknown.
NCCN^k^ guidelines (pre, broad, or NGS)	NCCN guidelines=NGS, NGS more likely.
Drug approval periods (1, 2, 3, 4, 5, 6, 7)	As drug approval periods increase, NGS more likely.
Laboratory values (high, normal, low, not tested) for alkaline phosphatase, alanine transaminase, aspartate transferase, bilirubin, creatine, lymphocyte count, red blood cell count, hematocrit, platelet count, white blood cell count, hemoglobin	The direction of a single laboratory value is unknown. However, generally one would expect multiple out-of-range values to reflect poor health and may make NGS less likely, but the a priori assumed direction is unknown.

^a^WHO: World Health Organization.

^b^ECOG: Eastern Cooperative Oncology Group.

^c^EGFR: epidermal growth factor receptor.

^d^ROS1: c-ros oncogene 1.

^e^ALK: anaplastic lymphoma kinase.

^f^BRAF: V-Raf murine sarcoma viral oncogene homolog B.

^g^KRAS: Kirsten rat sarcoma virus.

^h^PD-L1: programmed death ligand 1.

^i^MAC: Medicare Administrative Contractor.

^j^MolDX: Molecular Diagnostics Services.

^k^NCCN: National Comprehensive Cancer Network.

### Statistical Analysis

Descriptive analyses were conducted to summarize available data and to understand the extent of missingness in the database. Categorical variables were assessed using a 1-sided chi-square test or Fisher exact test and continuous variables using a 2-sided *t* test. Missing values were imputed using the random forest missing data algorithm (impute.rfsrc function in R package *randomForestSRC*) [23].

**Figure 1:**
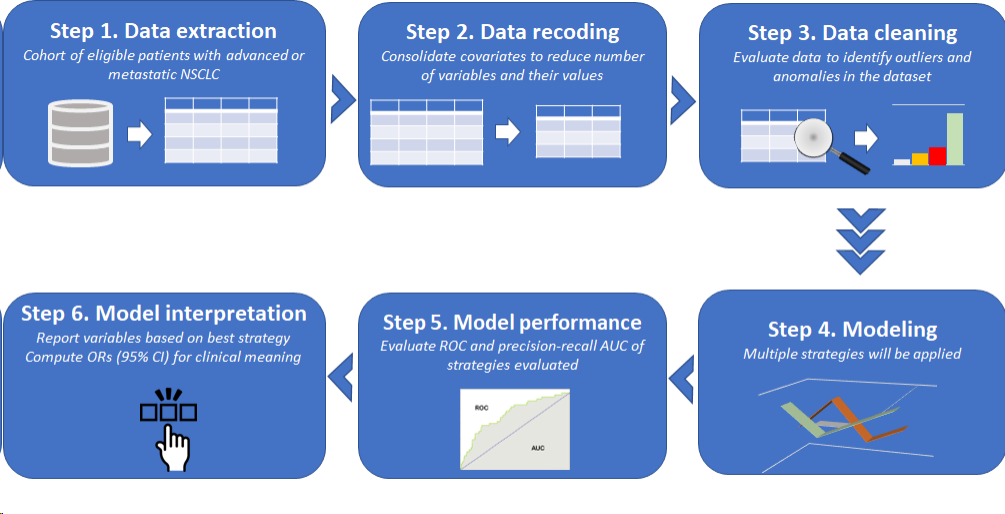
Data extraction and modeling flow. AUC: area under the curve; CI: confidence interval; NSCLC: non–small cell lung cancer; OR: odds ratio; ROC: receiver operating curve.

Three modeling strategies were used to identify potential predictors of NGS-based testing with 2 sets of outcomes for ever versus never NGS-tested (model 1) and early versus late NGS-tested (model 2). The 3 modeling strategies included logistic regression (LR) models, penalized logistic regression (PLR) using least absolute shrinkage and selection operator (LASSO) penalty, and extreme gradient boosting (XGBoost) with trees as base learners. LR was implemented using forward selection on the main effects and predefined interactions (listed earlier), starting with the predefined variables and adding the most significant terms to the model. PLR was implemented using sparse group LASSO on the main effects and predefined interactions, forcing some predefined variables into the model with the penalty selected using 5-fold cross-validation. XGBoost is a decision tree–based machine learning algorithm [24]. The model matrix for XGBoost was built using main effects and predefined interactions. Hyperparameters were selected based on 5-fold cross-validation over a grid search, and hyperparameters included the shrinkage (learning rate), the number of trees, and tree depth. Table S1 in Multimedia Appendix 1 contains the full list of hyperparameters used in this study. The data extraction approach and modeling process is summarized in Figure 1.

**Figure 2:**
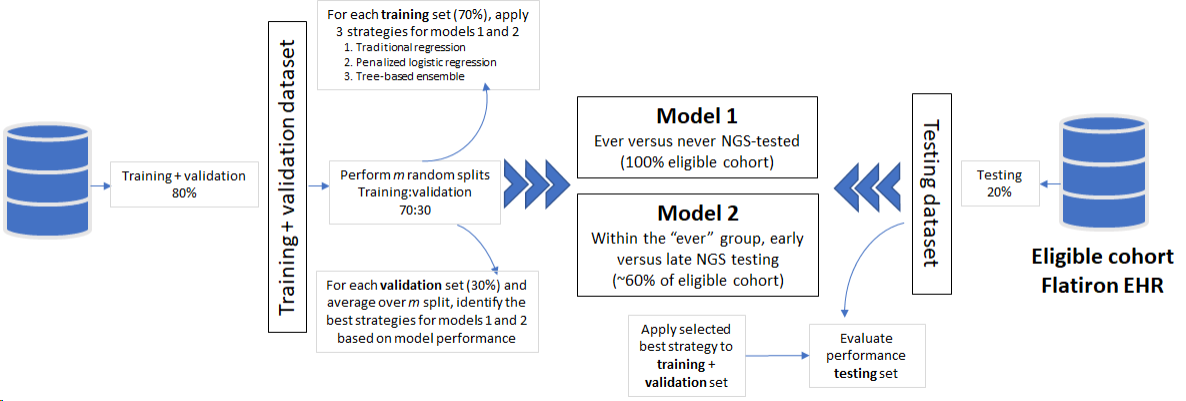
Modeling evaluation flow. EHR: electronic health record; NGS: next-generation sequencing.

In step 1, data were extracted based on the prespecified inclusion and exclusion criteria. Step 2 involved variable recoding, which included transforming all categorical variables with missing information by creating an additional level to represent missing data. Step 3 was a data quality method used to identify any unusual observations that needed to be excluded or recoded in addition to any imputation that was required. Steps 4 to 6 outline the implementation of models, evaluation of the performance of these models, and interpretation of the final features selected using LR. Figure 2 provides an overview of the model strategy evaluation process for the 2 outcomes mentioned in step 4 of Figure 1.

First, the data were split into D1 (training+validation; 80%) and D2 (testing; 20%) sets. Then, the 3 strategies were evaluated by comparing their performance on multiple *m*=1000 splits in the training (70%) and validation data (30%) within the D1 set. Specifically, for each split, all 3 strategies were fit to training data, and performance measures (eg, area under the receiver operating curve) were computed on the validation data. Modeling was done using R packages, sparsegl was used for LASSO, XGBoost for gradient boosting, and PRROC, which computes the areas under the precision-recall and ROC curve, for performance measures. PLR and XGBoost involved hyperparameters that were fine-tuned using 5-fold cross-validation nested within training datasets. Prediction models were developed on 2 different groups: ever versus never and early versus late NGS-tested groups. In total, 146 features (including all levels of all variables) were entered into both the XGBoost and LASSO models, with only 36 features (main effects and interactions) being used in the LR model. Preselection of features consisted of excluding variables that have little to no association with the outcomes of interest.

The final model was selected by evaluating performance as described earlier (area under the receiver operating curve from validation data) and by considering the simplicity and clinical interpretability. Model performance was re-estimated using the test data D2. For the final model choice, the features with nonzero coefficients selected by PLR were run on the D1 data. These variables were fitted to an LR model within the test data D2 to calculate model estimates (odds ratios, 95% CIs, and *P* values). Odds ratios for main effects in the presence of interaction terms were calculated using the analytical formula presented in Multimedia Appendix 2. All analyses were conducted using SAS (version 9.4; SAS Institute Inc) and R (version 4.0.3; R Foundation for Statistical Computing).

### Ethical Considerations

The data used for this study are deidentified and subject to obligations to prevent reidentification and protect patient confidentiality, and as such are not considered human subjects research and are exempt from review in accordance with the US Code of Federal Regulations [16].

## Results

A total of 74,211 patient records were available in the Flatiron Health NSCLC dataset for this analysis. After applying eligibility criteria, a total of 31,407 patients were included in this analysis. Of all patients, 42.75% (n=13,425) were included in the ever NGS-tested group and 57.25% (n=17,982) were included in the never NGS-tested group. Among those in the ever NGS-tested group, 84.08% (n=11,289) were early NGS-tested, and 15.91% (n=2136) late NGS-tested. Characteristics of these groups and subgroups used as features in the machine learning models are listed in Tables 2-11.

Most features were significantly different between both the ever and never NGS-tested as well as the early NGS versus late NGS-tested groups. Of note, smoking rates and testing conducted during the NCCN prerecommendations period were lower for the ever NGS-tested group (n=10,589, 78.88% vs n=14,987, 83.34% and n=2663, 19.84% vs n=10,734, 59.69%, respectively), and ECOG status of 0 (n=4410, 32.85% vs n=4665, 25.94%) was higher for the ever NGS-tested group versus those who were never tested. Similarly, for the early versus late NGS-tested groups, there was a higher proportion of patients with a history of smoking (n=9025, 79.95% vs n=1564, 73.22%) and a lower proportion of testing conducted during the NCCN prerecommendations period (n=1746, 15.47% vs n=917, 42.93%) as well as a lower proportion of ECOG status of 0 (n=3606, 31.94% vs n=804, 37.64%) for the early tested group.

Comparison of performance metrics for each model showed that the percent AUC was similar across models (80%-84% and 77%-80%) and marginally better when the models were fit on the ever versus never NGS-tested groups. In addition, other metrics were also comparable (Table S2 in Multimedia Appendix 1). The final model chosen was the LASSO model, as it was able to identify important features including interactions (those with nonzero coefficients after shrinkage) and the metrics for each model were highly comparable (Table S2 in Multimedia Appendix 1). Figures S1 and S2 in Multimedia Appendix 1 show the feature importance plots for both groups. The most important factors associated with ever versus never testing included year of diagnosis, observation of a PD-L1 test, Black or African American race, and number of single-gene tests observed. The most important factors associated with early versus late testing included the observation of a PD-L1 test, a positive single-gene test result, the year of diagnosis, and the geographical region of care. Later year of diagnosis, evidence of PD-L1 testing, patient race, positive single-gene test results, and region were among the top 5 predictors of NGS testing for both ever versus never as well as early versus late NGS testing.

**Table 2:** Demographic characteristics of the overall, ever, and never NGS^a^-tested study cohorts prior to imputation.

Characteristic			Overall (N=31,407)		Ever NGS-tested^b^ (n=13,425)		Never NGS-tested^c^ (n=17,982)		Ever NGS-tested versus never NGS-tested, *P* value^d^	
Age at initial diagnosis (years), mean (SD)			67.2 (9.8)		67.2 (10.1)		67.3 (9.5)		.66	
**Sex, n (%)**										.0007
	Female	16,680 (53.11)		7281 (54.23)		9399 (52.27)				
	Male	14,726 (46.89)		6144 (45.77)		8582 (47.73)				
	Unknown or missing	1 (0)		0 (0)		1 (0.01)				
**Race, n (%)**										<.0001
	Asian	1050 (3.34)		552 (4.11)		498 (2.77)				
	Black or African American	2845 (9.06)		1089 (8.11)		1756 (9.77)				
	White	21,248 (67.65)		9109 (67.85)		12,139 (67.51)				
	Other	3269 (10.41)		1392 (10.37)		1877 (10.44)				
	Unknown or missing	2995 (9.54)		1283 (9.56)		1712 (9.52)				
**Smoking status, n (%)**										<.0001
	History of smoking	25,576 (81.43)		10,589 (78.88)		14,987 (83.34)				
	No history of smoking	5657 (18.01)		2826 (21.05)		2831 (15.74)				
	Unknown or missing	174 (0.55)		10 (0.07)		164 (0.91)				
**ECOG^e^ performance status, n (%)**										<.0001
	0	9075 (28.89)		4410 (32.85)		4665 (25.94)				
	1	11,215 (35.71)		5275 (39.29)		5940 (33.03)				
	2	3401 (10.83)		1393 (10.38)		2008 (11.17)				
	3	762 (2.43)		306 (2.28)		456 (2.54)				
	4	51 (0.16)		17 (0.13)		34 (0.19)				
	Unknown or missing	6903 (21.98)		2024 (15.08)		4879 (27.13)				

^a^NGS: next-generation sequencing.

^b^Patients in the overall study cohort with evidence of NGS-based biomarker testing in the database.

^c^Patients in the overall study cohort with no evidence of NGS-based biomarker testing.

^d^Two-sided *t* test for continuous variables; chi-square or Fisher exact test (where expected cell size <5) for categorical variables.

^e^ECOG: Eastern Cooperative Oncology Group.

**Table 3:** Biomarker status of the overall, ever, and never NGS^a^-tested study cohorts prior to imputation.

Characteristic		Overall (N=31,407)	Ever NGS-tested^b^ (n=13,425)	Never NGS-tested^c^ (n=17,982)	Ever NGS-tested vs never NGS-tested, *P* value^d^	
**Non-NGS–based (single gene) ALK^e^ status, n (%)**						<.0001
	Positive	617 (1.96)	253 (1.88)	364 (2.02)		
	Not positive	15,626 (49.75)	6278 (46.76)	9348 (51.99)		
	Not tested	15,164 (48.28)	6894 (51.35)	8270 (45.99)		
**Non-NGS–based (single gene) BRAF^f^ status, n (%)**						<.0001
	Positive	94 (0.30)	32 (0.24)	62 (0.34)		
	Not positive	3775 (12.02)	1729 (12.88)	2046 (11.38)		
	Not tested	27,538 (87.68)	11,664 (86.88)	15,874 (88.28)		
**Non-NGS–based (single gene) EGFR^g^ status, n (%)**						<.0001
	Positive	2822 (8.99)	928 (6.91)	1894 (10.53)		
	Not positive	12,312 (39.20)	3427 (25.53)	8885 (49.41)		
	Not tested	16,273 (51.81)	9070 (67.56)	7203 (40.06)		
**Non-NGS–based (single gene) KRAS^h^ status, n (%)**						<.0001
	Positive	1141 (3.63)	298 (2.22)	843 (4.69)		
	Not positive	2958 (9.42)	1082 (8.06)	1876 (10.43)		
	Not tested	27,308 (86.95)	12,045 (89.72)	15,263 (84.88)		
**Non-NGS–based (single gene) ROS1^i^ status, n (%)**						<.0001
	Positive	128 (0.41)	58 (0.43)	70 (0.39)		
	Not positive	9383 (29.88)	5011 (37.33)	4372 (24.31)		
	Not tested	21,896 (69.72)	8356 (62.24)	13,540 (75.30)		
**Non-NGS–based (single gene) MET^j^ status, n (%)**						<.0001
	Positive	7 (0.02)	3 (0.02)	4 (0.02)		
	Not positive	1965 (6.26)	1517 (11.30)	448 (2.49)		
	Not tested	29,435 (93.72)	11,905 (88.68)	17,530 (97.49)		
**Non-NGS–based (single gene) RET^k^ status, n (%)**						<.0001
	Positive	34 (0.11)	27 (0.20)	7 (0.04)		
	Not positive	2381 (7.58)	1679 (12.51)	702 (3.90)		
	Not tested	28,992 (92.31)	11,719 (87.29)	17,273 (96.06)		
**Non-NGS–based (single gene) NTRK^l^ status, n (%)**						<.0001
	Positive	2 (0.01)	1 (0.01)	1 (0.01)		
	Not positive	747 (2.38)	617 (4.60)	130 (0.72)		
	Not tested	30,658 (97.62)	12,807 (95.40)	17,851 (99.27)		
**Non-NGS–based (single gene) testing^m^, n (%)**						<.0001
	Any positive result observed	4795 (15.27)	1576 (11.74)	3219 (17.90)		
	Never tested	11,968 (38.11)	5661 (42.17)	6307 (35.07)		
	Tested, but no positive results observed	14,644 (46.63)	6188 (46.09)	8456 (47.02)		
**PD-L1^n^ status, n (%)**						<.0001
	Positive	1826 (5.81)	1289 (9.60)	537 (2.99)		
	Not positive	9988 (31.80)	6354 (47.33)	3634 (20.21)		
	Not tested	19,593 (62.38)	5782 (43.07)	13,811 (76.80)		
Single-gene tests received^m^, mean (SD)		2.1 (2.0)	2.3 (2.0)	2.0 (1.9)	<.0001	

^a^NGS: next-generation sequencing.

^b^Patients in the overall study cohort with evidence of NGS-based biomarker testing in the database.

^c^Patients in the overall study cohort with no evidence of NGS-based biomarker testing.

^d^Two-sided *t* test for continuous variables; chi-square or Fisher exact test (where expected cell size <5) for categorical variables.

^e^ALK: anaplastic lymphoma kinase.

^f^BRAF: V-Raf Murine Sarcoma Viral Oncogene Homolog B.

^g^EGFR: epidermal growth factor receptor.

^h^KRAS: Kirsten rat sarcoma virus.

^i^ROS1: c-ros oncogene 1.

^j^MET: mesenchymal epithelial transition.

^k^RET: rearranged during transfection.

^l^NTRK: neurotrophic tyrosine receptor kinase.

^m^Results are based on biomarkers ALK, BRAF, EGFR, KRAS, ROS1, MET, RET, and NTRK.

^n^PD-L1: programmed death ligand 1.

**Table 4:** Geographic and time characteristics of the overall, ever, and never NGS^a^-tested study cohorts prior to imputation.

Characteristic		Overall (N=31,407), n (%)	Ever NGS-tested^b^ (n=13,425), n (%)	Never NGS-tested^c^ (n=17,982), n (%)	Ever NGS-tested vs never NGS-tested, *P* value^d^	
**MAC^e^ region**						<.0001
	JE Noridian	2097 (6.68)	814 (6.06)	1283 (7.13)		
	JF Noridian	2476 (7.88)	1111 (8.28)	1365 (7.59)		
	J6 NGS	856 (2.73)	335 (2.50)	521 (2.90)		
	J5 WPS	603 (1.92)	235 (1.75)	368 (2.05)		
	J8 WPS	2025 (6.45)	1051 (7.83)	974 (5.42)		
	JK NGS	2459 (7.83)	1102 (8.21)	1357 (7.55)		
	JL Novitas	2817 (8.97)	1283 (9.56)	1534 (8.53)		
	JM Palmetto	2218 (7.06)	858 (6.39)	1360 (7.56)		
	J15 CGS	924 (2.94)	397 (2.96)	527 (2.93)		
	JJ Cahaba	4194 (13.35)	2049 (15.26)	2145 (11.93)		
	JH Novitas	6093 (19.40)	2176 (16.21)	3917 (21.78)		
	Unknown or missing	4645 (14.79)	2014 (15)	2631 (14.63)		
**MolDX^f^ Program**						<.0001
	Yes	14,294 (45.51)	6399 (47.66)	7895 (43.91)		
	No	12,468 (39.70)	5012 (37.33)	7456 (41.46)		
	Unknown or missing	4645 (14.79)	2014 (15)	2631 (14.63)		
**NCCN^g^ guideline period**						<.0001
	Prerecommendations	13,397 (42.66)	2663 (19.84)	10,734 (59.69)		
	Broad-based testing recommended	13,552 (43.15)	7339 (54.67)	6213 (34.55)		
	NGS-based testing recommended	4458 (14.19)	3423 (25.50)	1035 (5.76)		
**Timing of diagnosis by drug approval period**						<.0001
	Period 1	1223 (3.89)	96 (0.72)	1127 (6.27)		
	Period 2	12,850 (40.91)	2823 (21.03)	10,027 (55.76)		
	Period 3	4396 (14)	1868 (13.91)	2528 (14.06)		
	Period 4	4877 (15.53)	2724 (20.29)	2153 (11.97)		
	Period 5	4613 (14.69)	3224 (24.01)	1389 (7.72)		
	Period 6	2858 (9.10)	2216 (16.51)	642 (3.57)		
	Period 7	590 (1.88)	474 (3.53)	116 (0.65)		

^a^NGS: next-generation sequencing.

^b^Patients in the overall study cohort with evidence of NGS-based biomarker testing in the database.

^c^Patients in the overall study cohort with no evidence of NGS-based biomarker testing.

^d^Two-sided *t* test for continuous variables; chi-square or Fisher exact test (where expected cell size <5) for categorical variables.

^e^MAC: Medicare Administration Contractor.

^f^MolDX: Molecular Diagnostics Services.

^g^NCCN: National Comprehensive Cancer Network.

**Table 5:** Clinical care characteristics of the overall, ever, and never NGS^a^-tested study cohorts prior to imputation.

Characteristic			Overall (N=31,407)		Ever NGS-tested^b^ (n=13,425)		Never NGS-tested^c^ (n=17,982)		Ever NGS-tested vs never NGS-tested, *P* value^d^	
**Practice setting, n (%)**										<.0001
	Academic	3626 (11.55)		1783 (13.28)		1843 (10.25)				
	Community	27,781 (88.45)		11,642 (86.72)		16,139 (89.75)				
**Insurance type, n (%)**										<.0001
	Private+public	4301 (13.69)		1940 (14.45)		2361 (13.13)				
	Private only	7083 (22.55)		3601 (26.82)		3482 (19.36)				
	Public only	4037 (12.85)		1560 (11.62)		2477 (13.77)				
	Multiple types	8997 (28.65)		4066 (30.29)		4931 (27.42)				
	Unknown or missing	6989 (22.25)		2258 (16.82)		4731 (26.31)				
**Stage at initial diagnosis, n (%)**										<.0001
	0-I	2736 (8.71)		1208 (9)		1528 (8.50)				
	II	1453 (4.63)		671 (5)		782 (4.35)				
	III	5621 (17.90)		2227 (16.59)		3394 (18.87)				
	IV	20,929 (66.64)		9096 (67.75)		11,833 (65.80)				
	Unknown or missing	668 (2.13)		223 (1.66)		445 (2.47)				
**Year of index diagnosis, n (%)**										<.0001
	2011	1896 (6.04)		158 (1.18)		1738 (9.67)				
	2012	2402 (7.65)		229 (1.71)		2173 (12.08)				
	2013	2699 (8.59)		476 (3.55)		2223 (12.36)				
	2014	3054 (9.72)		664 (4.95)		2390 (13.29)				
	2015	3346 (10.65)		1136 (8.46)		2210 (12.29)				
	2016	3397 (10.82)		1372 (10.22)		2025 (11.26)				
	2017	3472 (11.05)		1708 (12.72)		1764 (9.81)				
	2018	3401 (10.83)		1966 (14.64)		1435 (7.98)				
	2019	3282 (10.45)		2293 (17.08)		989 (5.50)				
	2020	2777 (8.84)		2066 (15.39)		711 (3.95)				
	2021	1681 (5.35)		1357 (10.11)		324 (1.80)				
Practice volume^e^, mean (SD)			154.1 (143.6)		169.2 (156.0)		142.8 (132.5)		<.0001	
**BMI, n (%)**										<.0001
	Underweight	1373 (4.37)		597 (4.45)		776 (4.32)				
	Normal weight	10,593 (33.73)		4638 (34.55)		5955 (33.12)				
	Overweight	8897 (28.33)		4019 (29.94)		4878 (27.13)				
	Obese	6492 (20.67)		2920 (21.75)		3572 (19.86)				
	Unknown or missing	4052 (12.90)		1251 (9.32)		2801 (15.58)				
Body weight (kg), mean (SD)			75.0 (18.6)		75.3 (18.8)		74.8 (18.4)		.04	
Duration of follow-up (days), mean (SD)			704.8 (638.1)		735.1 (636.5)		682.2 (638.3)		<.0001	

^a^NGS: next-generation sequencing.

^b^Patients in the overall study cohort with evidence of NGS-based biomarker testing in the database.

^c^Patients in the overall study cohort with no evidence of NGS-based biomarker testing.

^d^Two-sided *t* test for continuous variables; chi-square or Fisher exact test (where expected cell size <5) for categorical variables.

^e^Number of patients with non-small cell lung cancer receiving care at the same practice per year.

**Table 6:** Laboratory values of the overall, ever, and never NGS^a^-tested study cohorts prior to imputation.

Characteristic			Overall (N=31,407), n (%)		Ever NGS-tested^b^ (n=13,425), n (%)		Never NGS-tested^c^ (n=17,982), n (%)		Ever NGS-tested vs never NGS-tested, *P* value^d^
**ALP^e^**									<.0001
	High	3550 (11.30)		1583 (11.79)		1967 (10.94)			
	Low	146 (0.46)		60 (0.45)		86 (0.48)			
	Normal	15,295 (48.70)		6805 (50.69)		8490 (47.21)			
	Not tested	12,416 (39.53)		4977 (37.07)		7439 (41.37)			
**ALT^f^**									<.0001
	High	1480 (4.71)		676 (5.04)		804 (4.47)			
	Low	850 (2.71)		384 (2.86)		466 (2.59)			
	Normal	16,606 (52.87)		7389 (55.04)		9217 (51.26)			
	Not tested	12,471 (39.71)		4976 (37.07)		7495 (41.68)			
**AST^g^**									<.0001
	High	1364 (4.34)		579 (4.31)		785 (4.37)			
	Low	1018 (3.24)		447 (3.33)		571 (3.18)			
	Normal	16,706 (53.19)		7479 (55.71)		9227 (51.31)			
	Not tested	12,319 (39.22)		4920 (36.65)		7399 (41.15)			
**Bilirubin**									<.0001
	High	461 (1.47)		212 (1.58)		249 (1.38)			
	Low	1200 (3.82)		545 (4.06)		655 (3.64)			
	Normal	16,014 (50.99)		7138 (53.17)		8876 (49.36)			
	Not tested	13,732 (43.72)		5530 (41.19)		8202 (45.61)			
**Creatinine**									<.0001
	High	2272 (7.23)		950 (7.08)		1322 (7.35)			
	Low	2143 (6.82)		965 (7.19)		1178 (6.55)			
	Normal	15,512 (49.39)		6917 (51.52)		8595 (47.80)			
	Not tested	11,480 (36.55)		4593 (34.21)		6887 (38.30)			
**Lymphocyte count**									<.0001
	High	435 (1.39)		162 (1.21)		273 (1.52)			
	Low	7325 (23.32)		3270 (24.36)		4055 (22.55)			
	Normal	12,238 (38.97)		5504 (41)		6734 (37.45)			
	Not tested	11,409 (36.33)		4489 (33.44)		6920 (38.48)			
**Red blood cell count**									<.0001
	High	371 (1.18)		135 (1.01)		236 (1.31)			
	Low	5751 (18.31)		2336 (17.40)		3415 (18.99)			
	Normal	12,350 (39.32)		5551 (41.35)		6799 (37.81)			
	Not tested	12,935 (41.19)		5403 (40.25)		7532 (41.89)			
**Hematocrit**									<.0001
	High	482 (1.53)		187 (1.39)		295 (1.64)			
	Low	6440 (20.50)		2772 (20.65)		3668 (20.40)			
	Normal	13,085 (41.66)		6026 (44.89)		7059 (39.26)			
	Not tested	11,400 (36.30)		4440 (33.07)		6960 (38.71)			
**Platelet count**									.003
	High	2605 (8.29)		1038 (7.73)		1567 (8.71)			
	Low	675 (2.15)		271 (2.02)		404 (2.25)			
	Normal	14,807 (47.15)		6436 (47.94)		8371 (46.55)			
	Not tested	13,320 (42.41)		5680 (42.31)		7640 (42.49)			
**White blood cell count**									.03
	High	5171 (16.46)		2166 (16.13)		3005 (16.71)			
	Low	461 (1.47)		195 (1.45)		266 (1.48)			
	Normal	13,237 (42.15)		5790 (43.13)		7447 (41.41)			
	Not tested	12,538 (39.92)		5274 (39.28)		7264 (40.40)			
**Hemoglobin, whole blood**									<.0001
	High	406 (1.29)		141 (1.05)		265 (1.47)			
	Low	6973 (22.20)		2969 (22.12)		4004 (22.27)			
	Normal	13,193 (42.01)		5997 (44.67)		7196 (40.02)			
	Not tested	10,835 (34.50)		4318 (32.16)		6517 (36.24)			

^a^NGS: next-generation sequencing.

^b^Patients in the overall study cohort with evidence of NGS-based biomarker testing in the database.

^c^Patients in the overall study cohort with no evidence of NGS-based biomarker testing.

^d^Two-sided *t* test for continuous variables; chi-square or Fisher exact test (where expected cell size <5) for categorical variables.

^e^ALP: alkaline phosphatase.

^f^ALT: alanine transaminase.

^g^AST: aspartate aminotransferase.

**Table 7:** Demographic characteristics of early and late NGS^a^-tested study cohorts prior to imputation.

Characteristic		Early NGS-tested^b^ (n=11,289)	Late NGS-tested^c^ (n=2136)	Early NGS-tested vs late NGS-tested, *P* value^d^	
Age at initial diagnosis (years), mean (SD)		67.5 (10.1)	65.5 (10.0)	<.0001	
**Sex, n (%)**					.02
	Female	6073 (53.80)	1208 (56.55)		
	Male	5216 (46.20)	928 (43.45)		
**Race, n (%)**					<.0001
	Asian	408 (3.61)	144 (6.74)		
	Black or African American	897 (7.95)	192 (8.99)		
	White	7655 (67.81)	1454 (68.07)		
	Other	1215 (10.76)	177 (8.29)		
	Unknown or missing	1114 (9.87)	169 (7.91)		
**Smoking status, n (%)**					<.0001
	History of smoking	9025 (79.95)	1564 (73.22)		
	No history of smoking	2256 (19.98)	570 (26.69)		
	Unknown or missing	8 (0.07)	2 (0.09)		
**ECOG^e^ performance status, n (%)**					<.0001
	0	3606 (31.94)	804 (37.64)		
	1	4440 (39.33)	835 (39.09)		
	2	1220 (10.81)	173 (8.10)		
	3	282 (2.50)	24 (1.12)		
	4	15 (0.13)	2 (0.09)		
	Unknown or missing	1726 (15.29)	298 (13.95)		

^a^NGS: next-generation sequencing.

^b^Patients in the ever NGS-tested group whose first or only NGS-based test occurred prior to the start of first-line therapy through day 7 of first-line therapy.

^c^Patients in the ever NGS-tested group whose first NGS-based test occurred 8 days or later after the start of first-line therapy.

^d^Two-sided *t* test for continuous variables; chi-square or Fisher exact test (where expected cell size <5) for categorical variables.

^e^ECOG: Eastern Cooperative Oncology Group.

**Table 8:** Biomarker status of early and late NGS^a^-tested study cohorts prior to imputation.

Characteristic		Early NGS-tested^b^ (n=11,289)	Late NGS-tested^c^ (n=2136)	Early NGS-tested vs late NGS-tested, *P* value^d^	
**Non-NGS–based (single gene) ALK^e^ status, n (%)**					<.0001
	Positive	193 (1.71)	60 (2.81)		
	Not positive	5208 (46.13)	1070 (50.09)		
	Not tested	5888 (52.16)	1006 (47.10)		
**Non-NGS–based (single gene) BRAF^f^ status, n (%)**					.04
	Positive	25 (0.22)	7 (0.33)		
	Not positive	1420 (12.58)	309 (14.47)		
	Not tested	9844 (87.20)	1820 (85.21)		
**Non-NGS–based (single gene) EGFR^g^ status, n (%)**					<.0001
	Positive	435 (3.85)	493 (23.08)		
	Not positive	2589 (22.93)	838 (39.23)		
	Not tested	8265 (73.21)	805 (37.69)		
**Non-NGS–based (single gene) KRAS^h^ status, n (%)**					<.0001
	Positive	221 (1.96)	77 (3.60)		
	Not positive	825 (7.31)	257 (12.03)		
	Not tested	10,243 (90.73)	1802 (84.36)		
**Non-NGS–based (single gene) ROS1^i^ status, n (%)**					<.0001
	Positive	44 (0.39)	14 (0.66)		
	Not positive	4376 (38.76)	635 (29.73)		
	Not tested	6869 (60.85)	1487 (69.62)		
**Non-NGS–based (single gene) MET^j^ status, n (%)**					<.0001
	Positive	2 (0.02)	1 (0.05)		
	Not positive	1449 (12.84)	68 (3.18)		
	Not tested	9838 (87.15)	2067 (96.77)		
**Non-NGS–based (single gene) RET^k^ status, n (%)**					<.0001
	Positive	27 (0.24)	0 (0)		
	Not positive	1558 (13.80)	121 (5.66)		
	Not tested	9704 (85.96)	2015 (94.34)		
**Non-NGS–based (single gene) NTRK^l^ status, n (%)**					<.0001
	Positive	1 (0.01)	0 (0)		
	Not positive	596 (5.28)	21 (0.98)		
	Not tested	10,692 (94.71)	2115 (99.02)		
**Non-NGS–based (single gene) testing^m^, n (%)**					<.0001
	Any positive result observed	931 (8.25)	645 (30.20)		
	Never tested	4959 (43.93)	702 (32.87)		
	Tested, but no positive results observed	5399 (47.83)	789 (36.94)		
**PD-L1^n^ status, n (%)**					<.0001
	Positive	1228 (10.88)	61 (2.86)		
	Not positive	5785 (51.24)	569 (26.64)		
	Not tested	4276 (37.88)	1506 (70.51)		
Number of single-gene tests received^m^, mean (SD)		2.3 (2.1)	2.2 (2.0)	.002	

^a^NGS: next-generation sequencing.

^b^Patients in the ever NGS-tested group whose first or only NGS-based test occurred prior to the start of first-line therapy through day 7 of first-line therapy.

^c^Patients in the ever NGS-tested group whose first NGS-based test occurred 8 days or later after the start of first-line therapy.

^d^Two-sided *t* test for continuous variables; chi-square or Fisher exact test (where expected cell size <5) for categorical variables.

^e^ALK: anaplastic lymphoma kinase.

^f^BRAF: V-Raf murine sarcoma viral oncogene homolog B.

^g^EGFR: epidermal growth factor receptor.

^h^KRAS: Kirsten rat sarcoma virus.

^i^ROS1: c-ros oncogene 1.

^j^MET: mesenchymal epithelial transition.

^k^RET: rearranged during transfection.

^l^NTRK: neurotrophic tyrosine receptor kinase.

^m^Results are based on biomarkers ALK, BRAF, EGFR, KRAS, ROS1, MET, RET, and NTRK.

^n^PD-L1: programmed death ligand 1.

**Table 9:** Geographic and time characteristics of early and late NGS^a^-tested study cohorts prior to imputation.

Characteristic		Early NGS-tested^b^ (n=11,289), n (%)	Late NGS-tested^c^ (n=2136), n (%)	Early NGS-tested vs late NGS-tested, *P* value^d^	
**MAC^e^ region**					<.0001
	JE Noridian	639 (5.66)	175 (8.19)		
	JF Noridian	956 (8.47)	155 (7.26)		
	J6 NGS	283 (2.51)	52 (2.43)		
	J5 WPS	205 (1.82)	30 (1.40)		
	J8 WPS	921 (8.16)	130 (6.09)		
	JK NGS	924 (8.18)	178 (8.33)		
	JL Novitas	1094 (9.69)	189 (8.85)		
	JM Palmetto	707 (6.26)	151 (7.07)		
	J15 CGS	339 (3)	58 (2.72)		
	JJ Cahaba	1734 (15.36)	315 (14.75)		
	JH Novitas	1786 (15.82)	390 (18.26)		
	Unknown or missing	1701 (15.07)	313 (14.65)		
**MolDX^f^ Program**					.38
	Yes	5402 (47.85)	997 (46.68)		
	No	4186 (37.08)	826 (38.67)		
	Unknown or missing	1701 (15.07)	313 (14.65)		
**NCCN^g^ guideline period**					<.0001
	Pre recommendations	1746 (15.47)	917 (42.93)		
	Broad-based testing recommended	6286 (55.68)	1053 (49.30)		
	NGS-based testing recommended	3257 (28.85)	166 (7.77)		
**Timing of diagnosis by drug approval period**					<.0001
	Period 1	43 (0.38)	53 (2.48)		
	Period 2	1902 (16.85)	921 (43.12)		
	Period 3	1458 (12.92)	410 (19.19)		
	Period 4	2347 (20.79)	377 (17.65)		
	Period 5	2955 (26.18)	269 (12.59)		
	Period 6	2122 (18.80)	94 (4.40)		
	Period 7	462 (4.09)	12 (0.56)		

^a^NGS: next-generation sequencing.

^b^Patients in the ever NGS-tested group whose first or only NGS-based test occurred prior to the start of first-line therapy through day 7 of first-line therapy.

^c^Patients in the ever NGS-tested group whose first NGS-based test occurred 8 days or later after the start of first-line therapy.

^d^Two-sided *t* test for continuous variables; chi-square or Fisher exact test (where expected cell size <5) for categorical variables.

^e^MAC: Medicare Administration Contractor.

^f^MolDX: Molecular Diagnostics Services.

^g^NCCN: National Comprehensive Cancer Network.

**Table 10:** Clinical care characteristics of early and late NGS^a^-tested study cohorts prior to imputation.

Characteristic		Early NGS-tested^b^ (n=11,289)	Late NGS-tested^c^ (n=2136)	Early NGS-tested vs late NGS-tested, *P* value^d^	
**Practice setting, n (%)**					.50
	Academic	1509 (13.37)	274 (12.83)		
	Community	9780 (86.63)	1862 (87.17)		
**Insurance type, n (%)**					<.0001
	Private+public	1689 (14.96)	251 (11.75)		
	Private only	3026 (26.80)	575 (26.92)		
	Public only	1317 (11.67)	243 (11.38)		
	Multiple types	3491 (30.92)	575 (26.92)		
	Unknown or missing	1766 (15.64)	492 (23.03)		
**Stage at initial diagnosis, n (%)**					.0004
	0-I	1051 (9.31)	157 (7.35)		
	II	580 (5.14)	91 (4.26)		
	III	1822 (16.14)	405 (18.96)		
	IV	7655 (67.81)	1441 (67.46)		
	Unknown or missing	181 (1.60)	42 (1.97)		
**Year of index diagnosis**					<.0001
	2011	69 (0.61)	89 (4.17)		
	2012	121 (1.07)	108 (5.06)		
	2013	295 (2.61)	181 (8.47)		
	2014	430 (3.81)	234 (10.96)		
	2015	831 (7.36)	305 (14.28)		
	2016	1038 (9.19)	334 (15.64)		
	2017	1425 (12.62)	283 (13.25)		
	2018	1712 (15.17)	254 (11.89)		
	2019	2111 (18.70)	182 (8.52)		
	2020	1939 (17.18)	127 (5.95)		
	2021	1318 (11.68)	39 (1.83)		
Practice volume^e^, mean (SD)		169.6 (156.7)	166.8 (152.4)	.44	
**BMI, n (%)**					<.0001
	Underweight	529 (4.69)	68 (3.18)		
	Normal weight	3963 (35.10)	675 (31.60)		
	Overweight	3410 (30.21)	609 (28.51)		
	Obese	2435 (21.57)	485 (22.71)		
	Unknown or missing	952 (8.43)	299 (14)		
Body weight (kg), mean (SD)		75.2 (18.8)	75.9 (18.7)	.11	
Duration of follow-up (days), mean (SD)		644.1 (547.5)	1216.2 (829.1)	<.0001	

^a^NGS: next-generation sequencing.

^b^Patients in the ever NGS-tested group whose first or only NGS-based test occurred prior to the start of first-line therapy through day 7 of first-line therapy.

^c^Patients in the ever NGS-tested group whose first NGS-based test occurred 8 days or later after the start of first-line therapy.

^d^Two-sided *t* test for continuous variables; chi-square or Fisher exact test (where expected cell size <5) for categorical variables.

^e^Number of patients with non-small cell lung cancer receiving care at the same practice per year.

**Table 11:** Laboratory values of early and late NGS^a^-tested study cohorts prior to imputation.

Characteristic		Early NGS-tested^b^ (n=11,289)	Late NGS-tested^c^ (n=2136)	Early NGS-tested vs late NGS-tested, *P* value^d^	
**ALP^e^**					.79
	High	1339 (11.86)	244 (11.42)		
	Low	48 (0.43)	12 (0.56)		
	Normal	5720 (50.67)	1085 (50.80)		
	Not tested	4182 (37.04)	795 (37.22)		
**ALT^f^**					.01
	High	577 (5.11)	99 (4.63)		
	Low	345 (3.06)	39 (1.83)		
	Normal	6192 (54.85)	1197 (56.04)		
	Not tested	4175 (36.98)	801 (37.50)		
**AST^g^**					.07
	High	486 (4.31)	93 (4.35)		
	Low	396 (3.51)	51 (2.39)		
	Normal	6278 (55.61)	1201 (56.23)		
	Not tested	4129 (36.58)	791 (37.03)		
**Bilirubin**					.47
	High	182 (1.61)	30 (1.40)		
	Low	470 (4.16)	75 (3.51)		
	Normal	5992 (53.08)	1146 (53.65)		
	Not tested	4645 (41.15)	885 (41.43)		
**Creatinine**					.52
	High	815 (7.22)	135 (6.32)		
	Low	813 (7.20)	152 (7.12)		
	Normal	5808 (51.45)	1109 (51.92)		
	Not tested	3853 (34.13)	740 (34.64)		
**Lymphocyte count**					.003
	High	122 (1.08)	40 (1.87)		
	Low	2792 (24.73)	478 (22.38)		
	Normal	4611 (40.85)	893 (41.81)		
	Not tested	3764 (33.34)	725 (33.94)		
**Red blood cell count**					.001
	High	108 (0.96)	27 (1.26)		
	Low	2004 (17.75)	332 (15.54)		
	Normal	4594 (40.69)	957 (44.80)		
	Not tested	4583 (40.60)	820 (38.39)		
**Hematocrit**					.02
	High	150 (1.33)	37 (1.73)		
	Low	2378 (21.06)	394 (18.45)		
	Normal	5031 (44.57)	995 (46.58)		
	Not tested	3730 (33.04)	710 (33.24)		
**Platelet count**					.04
	High	855 (7.57)	183 (8.57)		
	Low	233 (2.06)	38 (1.78)		
	Normal	5372 (47.59)	1064 (49.81)		
	Not tested	4829 (42.78)	851 (39.84)		
**White blood cell count**					.04
	High	1837 (16.27)	329 (15.40)		
	Low	162 (1.44)	33 (1.54)		
	Normal	4811 (42.62)	979 (45.83)		
	Not tested	4479 (39.68)	795 (37.22)		
**Hemoglobin, whole blood**					.0004
	High	111 (0.98)	30 (1.40)		
	Low	2564 (22.71)	405 (18.96)		
	Normal	4987 (44.18)	1010 (47.28)		
	Not tested	3627 (32.13)	691 (32.35)		

^a^NGS: next-generation sequencing.

^b^Patients in the ever NGS-tested group whose first or only NGS-based test occurred prior to the start of first-line therapy through day 7 of first-line therapy.

^c^Patients in the ever NGS-tested group whose first NGS-based test occurred 8 days or later after the start of first-line therapy.

^d^Two-sided *t* test for continuous variables; chi-square or Fisher exact test (where expected cell size <5) for categorical variables.

^e^ALP: alkaline phosphatase.

^f^ALT: alanine transaminase.

^g^AST: aspartate aminotransferase.

**Figure 3:**
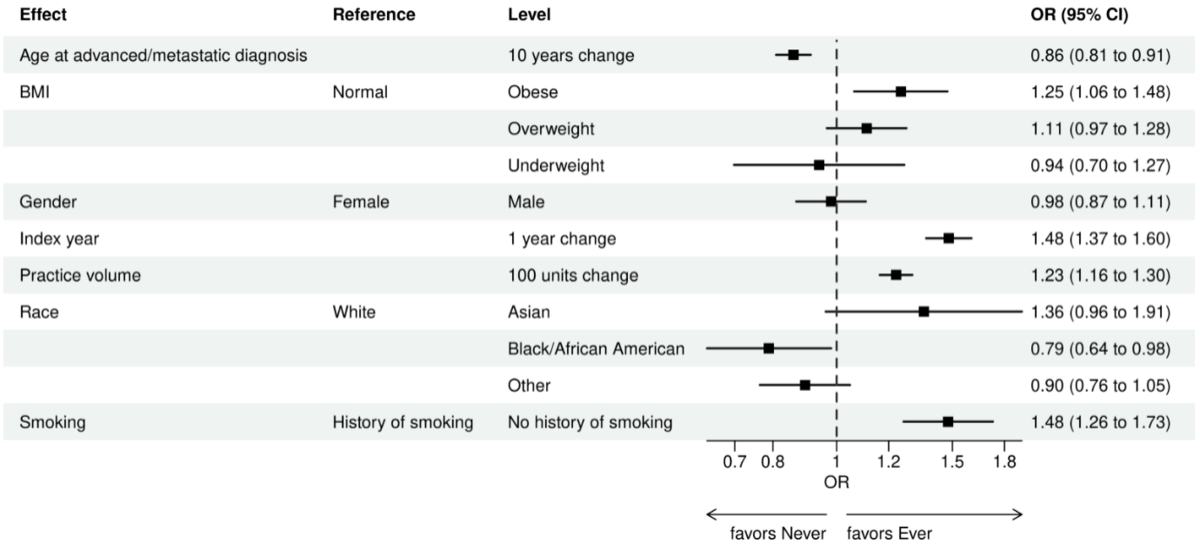
Forest plot of ever versus never NGS-tested: variables determined by a logistic regression model from variables preselected by a LASSO model: clinical care and demographic variables. Ever NGS-tested: patients in the overall study cohort with evidence of NGS-based biomarker testing in the database; never NGS-tested: patients in the overall study cohort with no evidence of NGS-based biomarker testing. Index year: year of index diagnosis; LASSO: least absolute shrinkage and selection operator; NGS: next-generation sequencing; OR: odds ratio.

**Figure 4:**
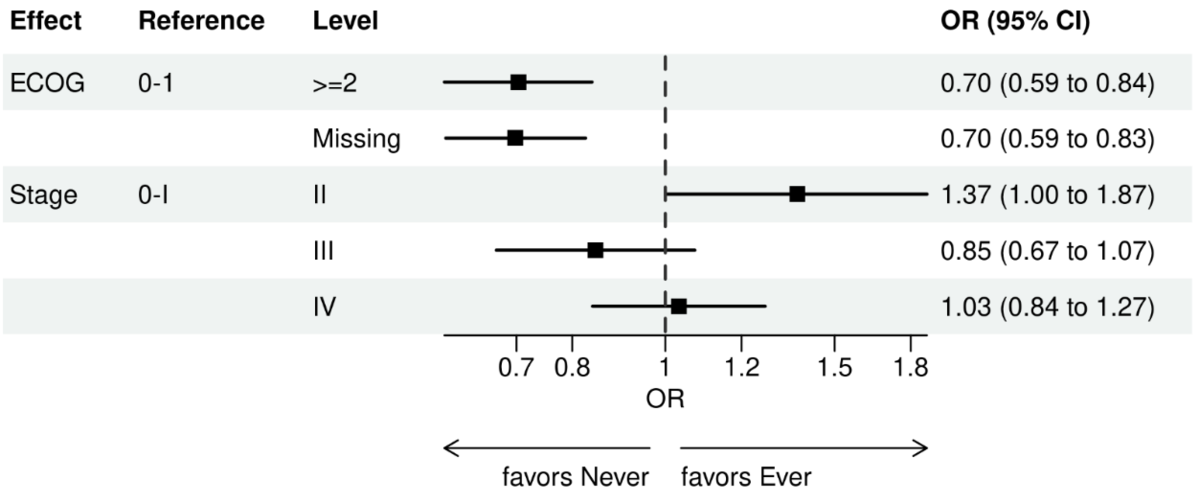
Forest plot of ever versus never NGS-tested: variables determined by a logistic regression model from variables preselected by a LASSO model: ECOG performance status and stage. Ever NGS-tested: patients in the overall study cohort with evidence of NGS-based biomarker testing in the database; never NGS-tested: patients in the overall study cohort with no evidence of NGS-based biomarker testing. ECOG: Eastern Cooperative Oncology Group; LASSO: least absolute shrinkage and selection operator; NGS: next-generation sequencing; OR: odds ratio.

**Figure 5:**
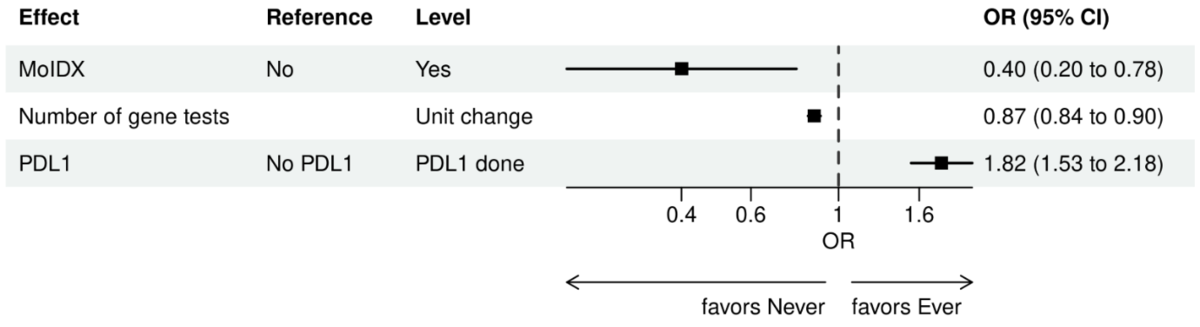
Forest plot of ever versus never NGS-tested: variables determined by a logistic regression model from variables preselected by a LASSO model: biomarkers and MolDX region. Ever NGS-tested: patients in the overall study cohort with evidence of NGS-based biomarker testing in the database; never NGS-tested: patients in the overall study cohort with no evidence of NGS-based biomarker testing. LASSO: least absolute shrinkage and selection operator; MolDX: Molecular Diagnostics Services; NGS: next-generation sequencing; OR: odds ratio; PDL1: programmed death ligand 1.

**Figure 6:**
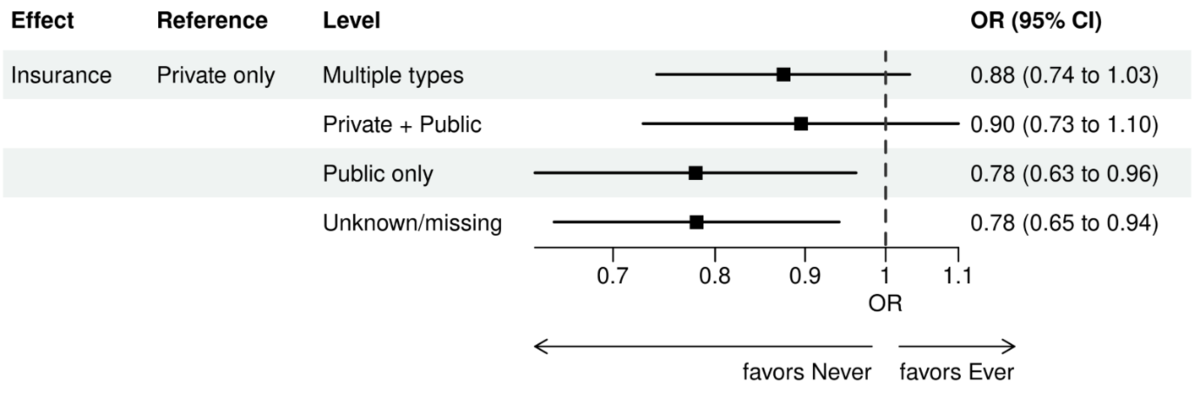
Forest plot of ever versus never NGS-tested: variables determined by a logistic regression model from variables preselected by a LASSO model: insurance. Ever NGS-tested: patients in the overall study cohort with evidence of NGS-based biomarker testing in the database; never NGS-tested: patients in the overall study cohort with no evidence of NGS-based biomarker testing. LASSO: least absolute shrinkage and selection operator; NGS: next-generation sequencing; OR: odds ratio.

**Figure 7:**
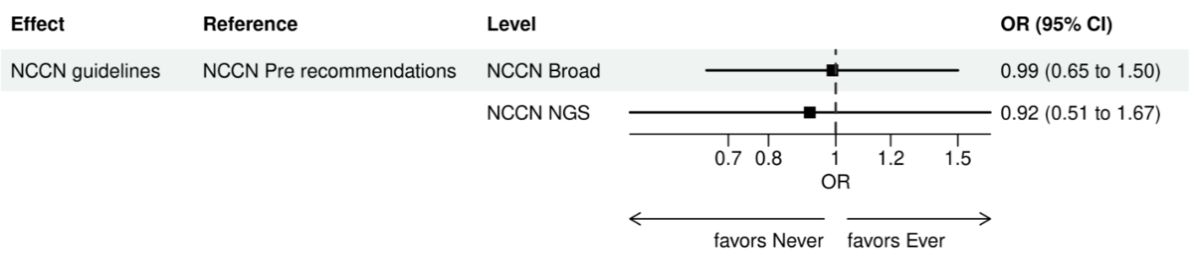
Forest plot of ever versus never NGS-tested: variables determined by a logistic regression model from variables preselected by a LASSO model: NCCN guidelines. Ever NGS-tested: patients in the overall study cohort with evidence of NGS-based biomarker testing in the database; never NGS-tested: patients in the overall study cohort with no evidence of NGS-based biomarker testing. LASSO: least absolute shrinkage and selection operator; NCCN: National Comprehensive Cancer Network; NGS: next-generation sequencing; OR: odds ratio.

**Figure 8:**
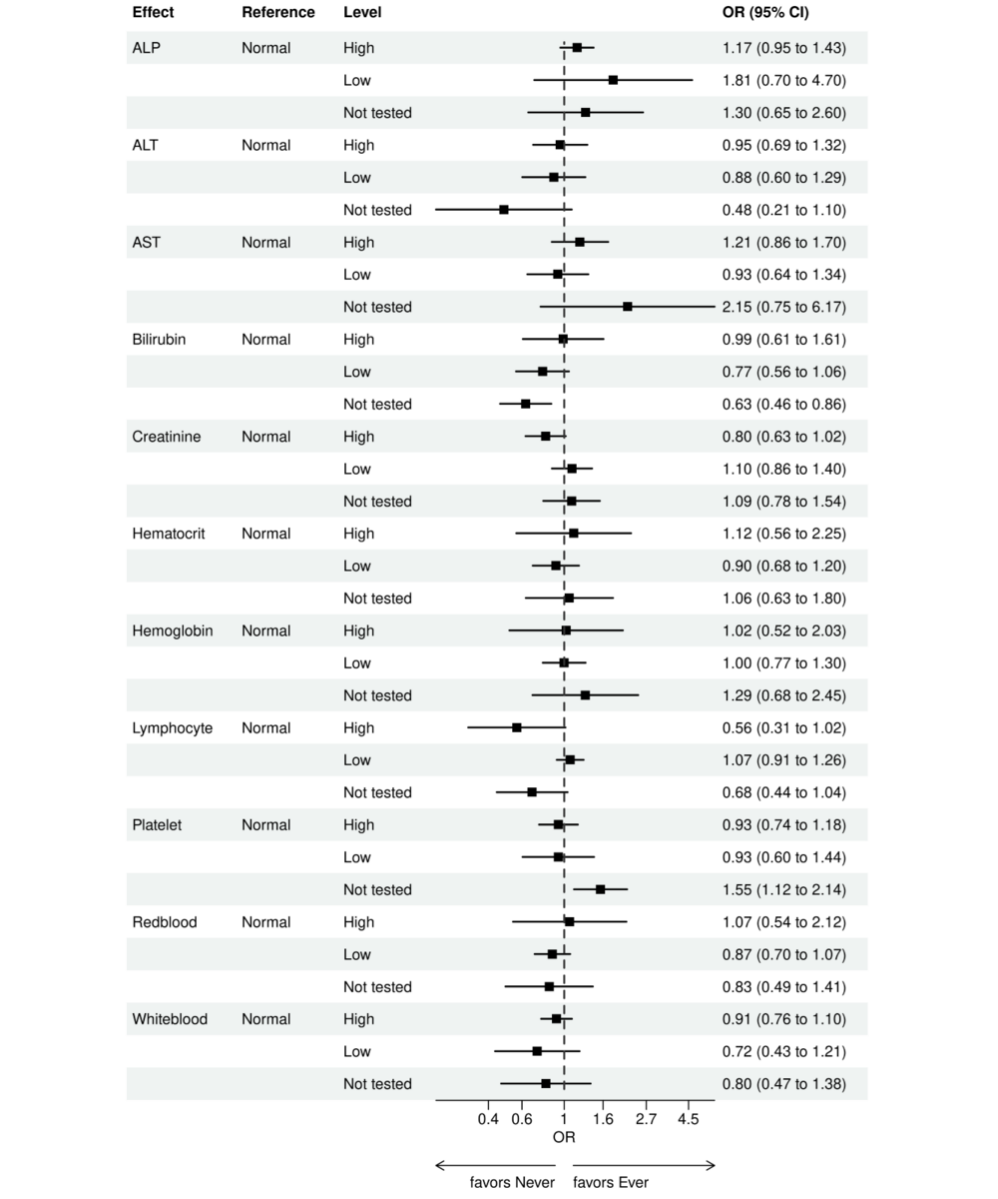
Forest plot of ever versus never NGS-tested: variables determined by a logistic regression model from variables preselected by a LASSO model: laboratory values. Ever NGS-tested: patients in the overall study cohort with evidence of NGS-based biomarker testing in the database; never NGS-tested: patients in the overall study cohort with no evidence of NGS-based biomarker testing. ALP: alkaline phosphatase; ALT: alanine transaminase; AST: aspartate aminotransferase; LASSO: least absolute shrinkage and selection operator; NGS: next-generation sequencing; OR: odds ratio.

**Figure 9:**
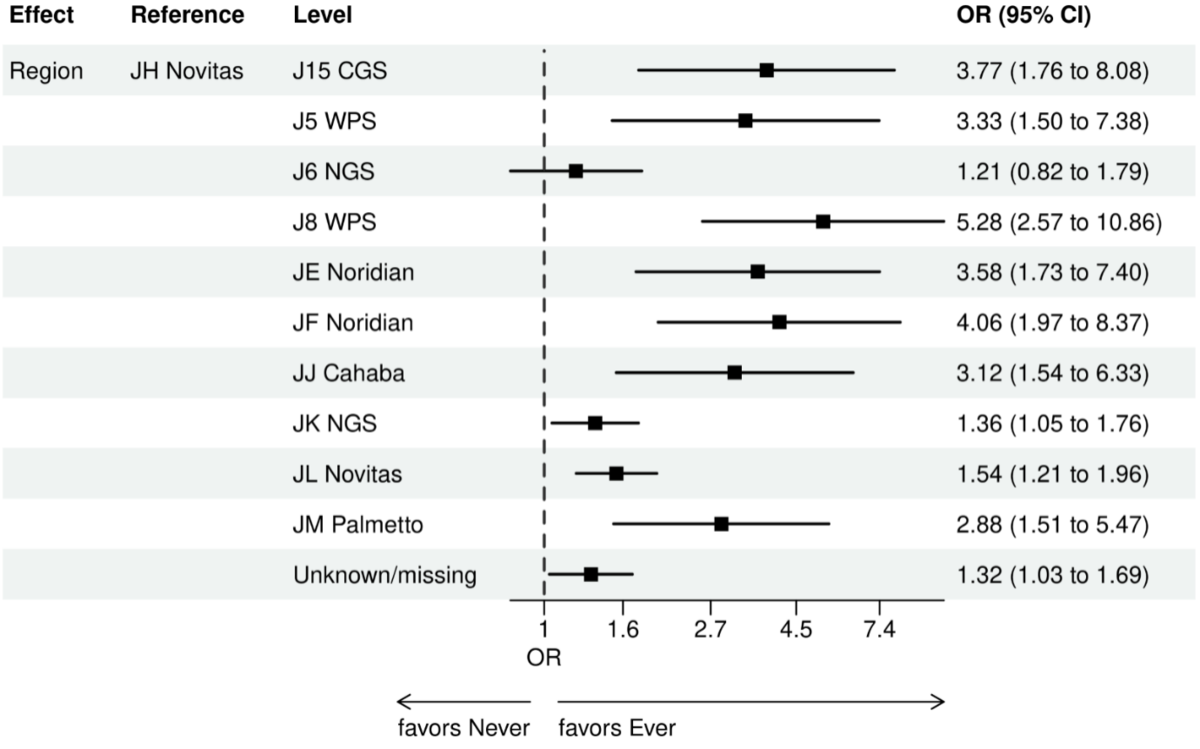
Forest plot of ever versus never NGS-tested: variables determined by a logistic regression model from variables preselected by a LASSO model: geographic region. Geographic regions reflect Medicare Administration Contractors. Ever NGS-tested: patients in the overall study cohort with evidence of NGS-based biomarker testing in the database; never NGS-tested: patients in the overall study cohort with no evidence of NGS-based biomarker testing. LASSO: least absolute shrinkage and selection operator; NGS: next-generation sequencing; OR: odds ratio.

Over the 1000 bootstrap samples over the training data D1, an average of 135 and 89 features were identified by the LASSO models for the ever versus never and early versus late NGS-tested groups, respectively. These variables were then entered into an LR model using the testing set. The final model was established after removing any nonsignificant interaction terms, as explained earlier in the study methods. Details of the model fit statistics are shown in Table S3 in Multimedia Appendix 1. All main effects identified from the modeling for each group are shown in Figures 3-9.

There were lower odds of ever receiving NGS testing among patients with later age at initial diagnosis, bilirubin not tested, worse ECOG performance status, treated in geographies under the MolDX Program, a total higher number of genetic tests received, had only public insurance, and who were of Black or African American race as compared with those who were never tested. Patients who were obese, had a later year of initial NSCLC diagnosis, were from larger practices, had evidence of PD-L1 testing, no results for platelet testing, no history of smoking, had stage II disease, and were treated in a MAC region other than JH Novitas or J6 NGS had higher odds of ever receiving NGS-based testing.

**Figure 10:**
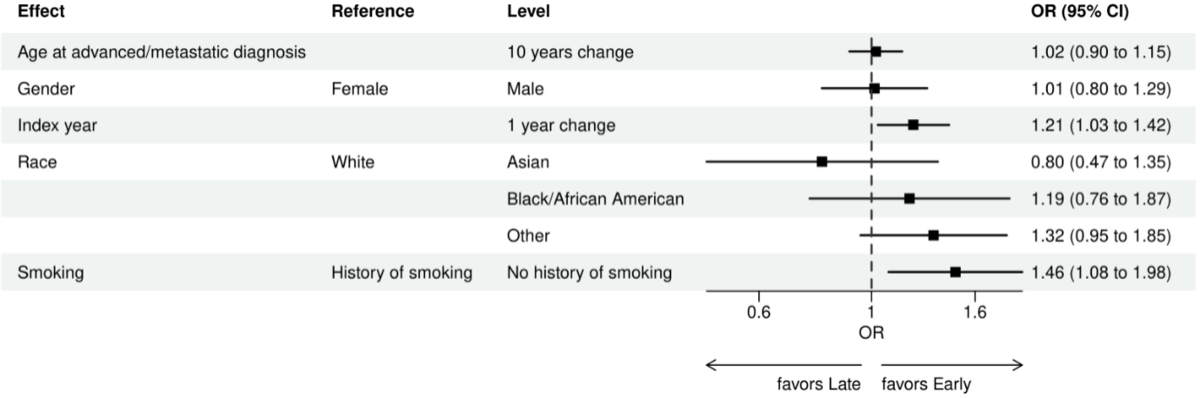
Forest plots early versus late NGS-tested: variables determined by logistic regression from variables preselected by a LASSO model: clinical care and demographic variables. Early NGS-tested: patients in the ever NGS-tested group whose first or only NGS-based test occurred prior to the start of first-line therapy through day 7 of first-line therapy; late NGS-tested: patients in the ever NGS-tested group whose first NGS-based test occurred 8 days or later after the start of first-line therapy. Index year: year of index diagnosis; LASSO: least absolute shrinkage and selection operator; NGS: next-generation sequencing; OR: odds ratio.

**Figure 11:**
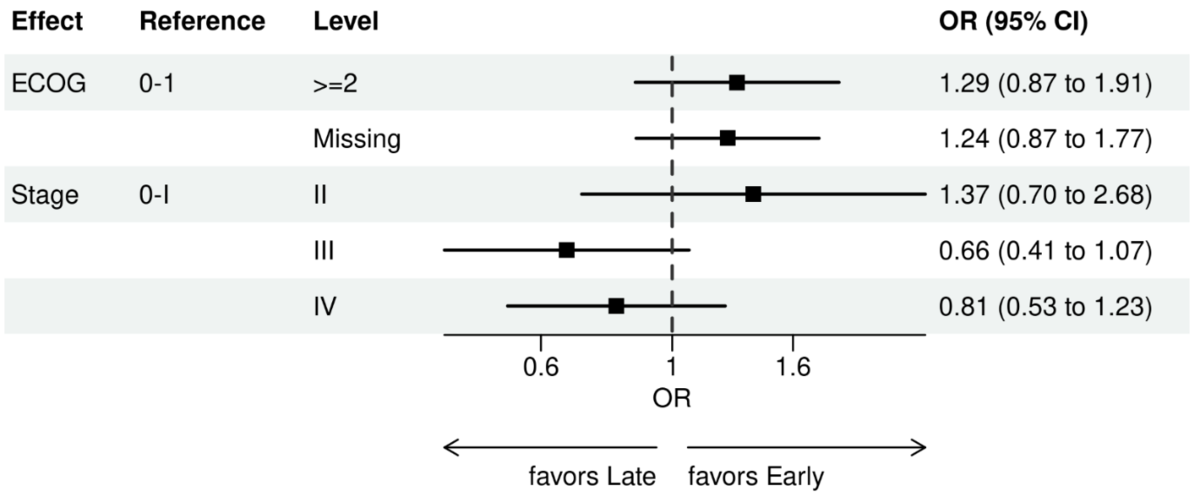
Forest plots early versus late NGS-tested: variables determined by logistic regression from variables preselected by a LASSO model: ECOG performance status and stage. Early NGS-tested: patients in the ever NGS-tested group whose first or only NGS-based test occurred prior to the start of first-line therapy through day 7 of first-line therapy; late NGS-tested: patients in the ever NGS-tested group whose first NGS-based test occurred 8 days or later after the start of first-line therapy. ECOG: Eastern Cooperative Oncology Group; LASSO: least absolute shrinkage and selection operator; NGS: next-generation sequencing; OR: odds ratio.

**Figure 12:**
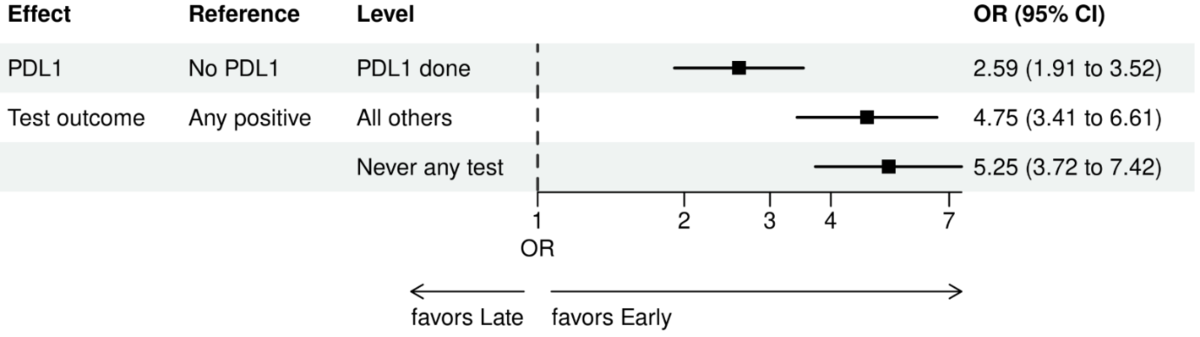
Forest plots early versus late NGS-tested: variables determined by logistic regression from variables preselected by a LASSO model: biomarkers. Early NGS-tested: patients in the ever NGS-tested group whose first or only NGS-based test occurred prior to the start of first-line therapy through day 7 of first-line therapy; late NGS-tested: patients in the ever NGS-tested group whose first NGS-based test occurred 8 days or later after the start of first-line therapy. LASSO: least absolute shrinkage and selection operator; NGS: next-generation sequencing; OR: odds ratio; PDL1: programmed death ligand 1.

**Figure 13:**
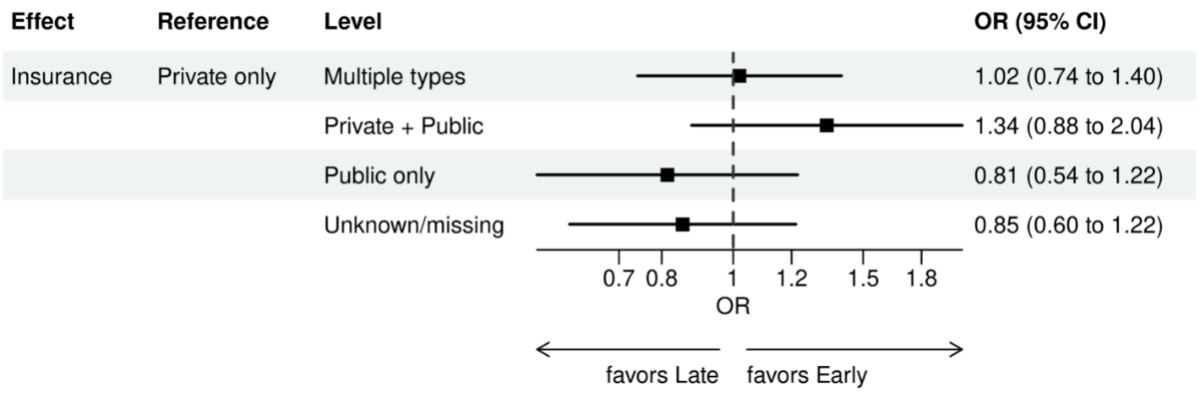
Forest plots early versus late NGS-tested: variables determined by logistic regression from variables preselected by a LASSO model: insurance. Early NGS-tested: patients in the ever NGS-tested group whose first or only NGS-based test occurred prior to the start of first-line therapy through day 7 of first-line therapy; late NGS-tested: patients in the ever NGS-tested group whose first NGS-based test occurred 8 days or later after the start of first-line therapy. LASSO: least absolute shrinkage and selection operator; NGS: next-generation sequencing; OR: odds ratio.

**Figure 14:**
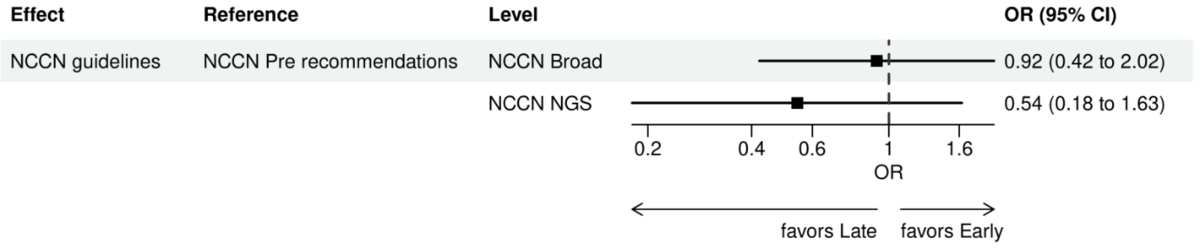
Forest plots early versus late NGS-tested: variables determined by logistic regression from variables preselected by a LASSO model: NCCN guidelines. Early NGS-tested: patients in the ever NGS-tested group whose first or only NGS-based test occurred prior to the start of first-line therapy through day 7 of first-line therapy; late NGS-tested: patients in the ever NGS-tested group whose first NGS-based test occurred 8 days or later after the start of first-line therapy. LASSO: least absolute shrinkage and selection operator; NCCN: National Comprehensive Cancer Network; NGS: next-generation sequencing; OR: odds ratio.

**Figure 15:**
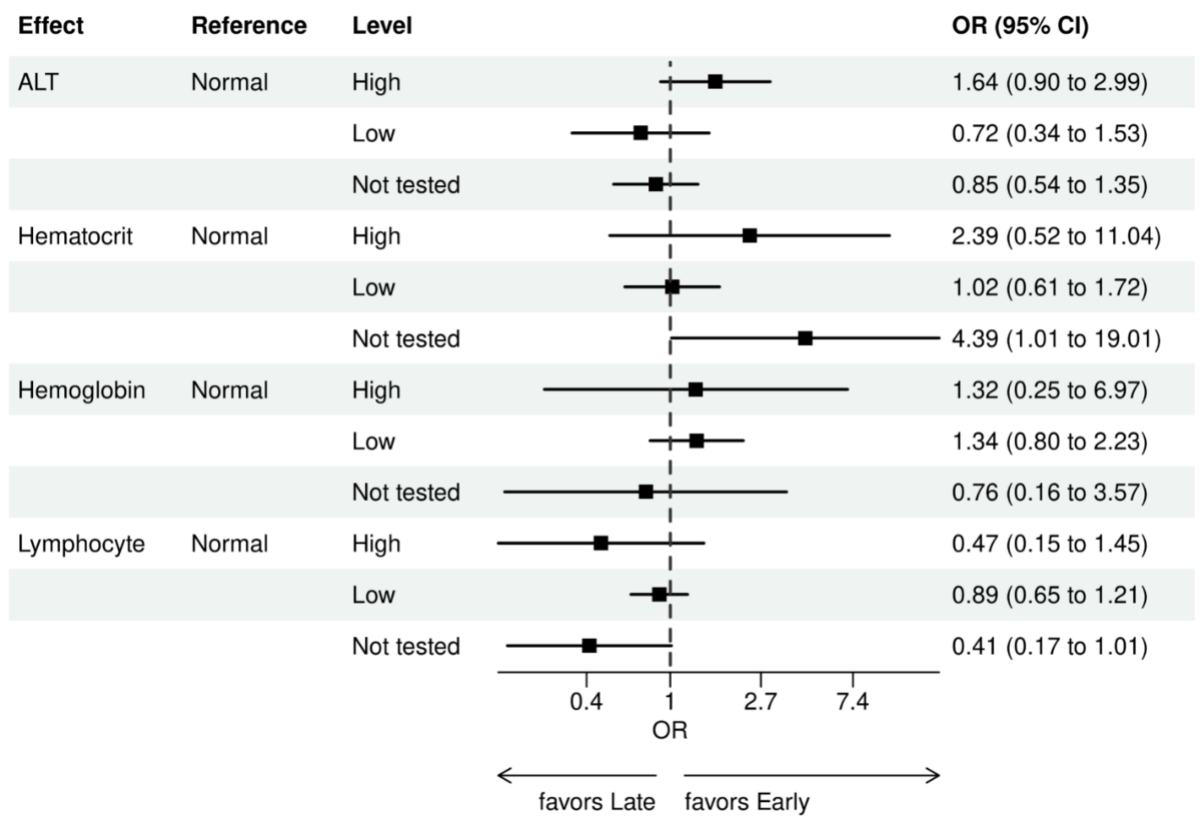
Forest plots early versus late NGS-tested: variables determined by logistic regression from variables preselected by a LASSO model: laboratory values. Early NGS-tested: patients in the ever NGS-tested group whose first or only NGS-based test occurred prior to the start of first-line therapy through day 7 of first-line therapy; late NGS-tested: patients in the ever NGS-tested group whose first NGS-based test occurred 8 days or later after the start of first-line therapy. ALT: alanine transaminase; LASSO: least absolute shrinkage and selection operator; NGS: next-generation sequencing; OR: odds ratio.

**Figure 16:**
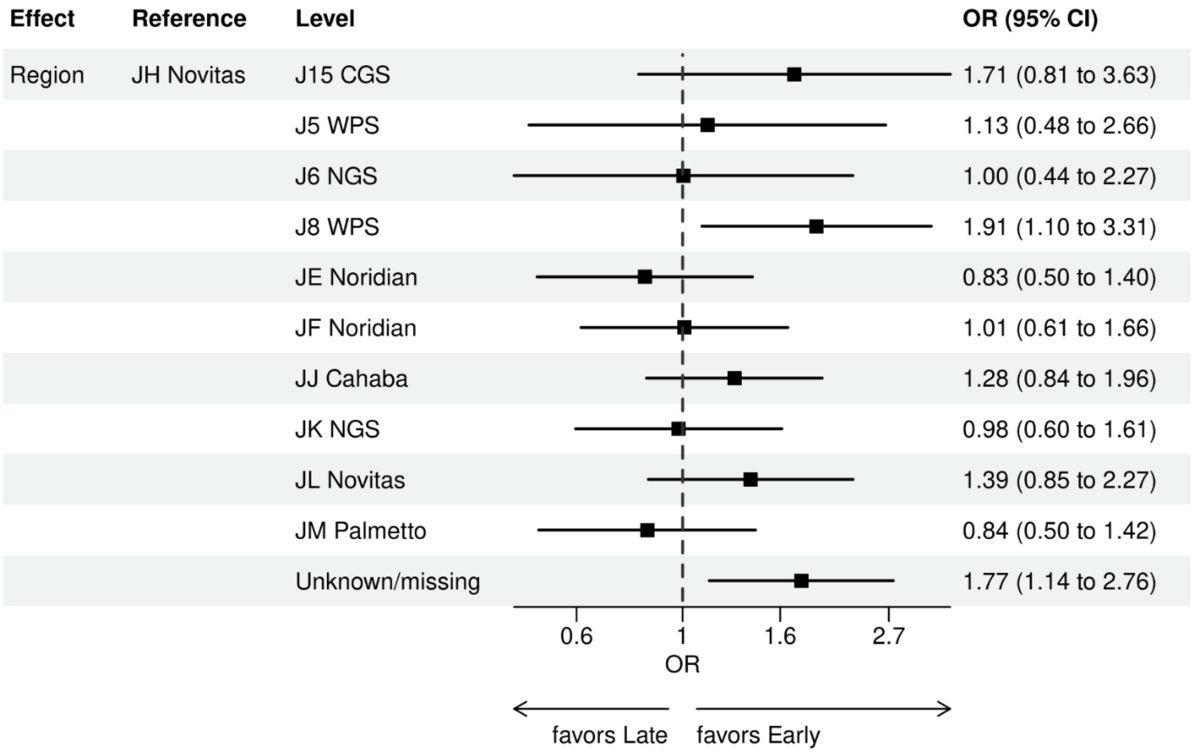
Forest plots early versus late NGS-tested: variables determined by logistic regression from variables preselected by a LASSO model: geographic region. Geographic regions reflect Medicare Administration Contractors. Early NGS-tested: patients in the ever NGS-tested group whose first or only NGS-based test occurred prior to the start of first-line therapy through day 7 of first-line therapy; late NGS-tested: patients in the ever NGS-tested group whose first NGS-based test occurred 8 days or later after the start of first-line therapy. LASSO: least absolute shrinkage and selection operator; NGS: next-generation sequencing; OR: odds ratio.

**Figure 17:**
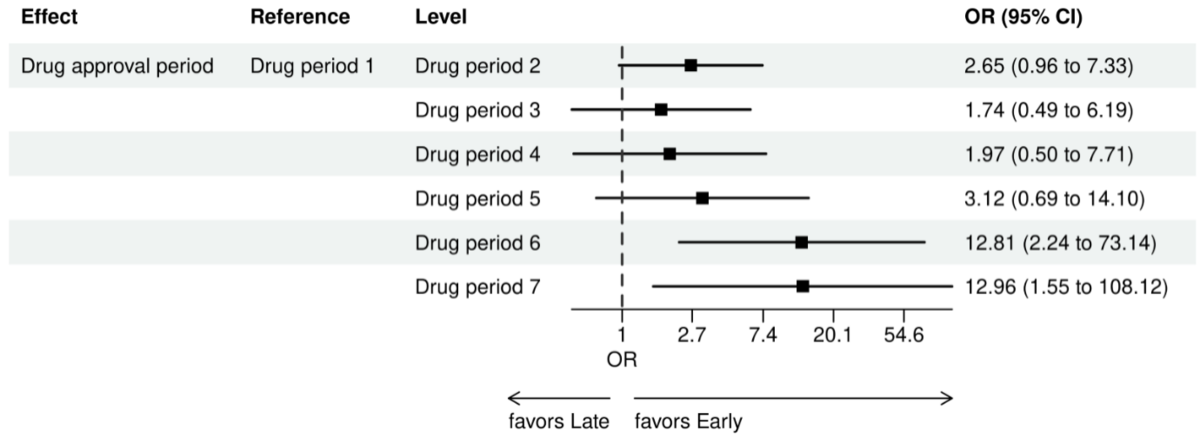
Forest plots early versus late NGS-tested: variables determined by logistic regression from variables preselected by a LASSO model: time period variables. Early NGS-tested: patients in the ever NGS-tested group whose first or only NGS-based test occurred prior to the start of first-line therapy through day 7 of first-line therapy; late NGS-tested: patients in the ever NGS-tested group whose first NGS-based test occurred 8 days or later after the start of first-line therapy. ALK: anaplastic lymphoma kinase; BRAF: V-Raf murine sarcoma viral oncogene homolog B; EGFR: epidermal growth factor receptor; KRAS: Kirsten rat sarcoma virus; LASSO: least absolute shrinkage and selection operator; MET: mesenchymal epithelial transition; NGS: next-generation sequencing; NTRK: neurotrophic tyrosine receptor kinase; OR: odds ratio; RET: rearranged during transfection; ROS1: c-ros oncogene 1; period 1: January 1-August 25, 2011 (EGFR drugs only); period 2: August 26, 2011-March 10, 2016 (EGFR+ALK); period 3: March 11, 2016-June 21, 2017 (EGFR+ALK+ROS1); period 4: June 22, 2017-November 25, 2018 (EGFR+ALK+ROS1+BRAF); period 5: November 26, 2018-May 5, 2020 (EGFR+ALK+ROS1+BRAF+NTRK); period 6: May 6, 2020-May 26, 2021 (EGFR+ALK+ROS1+BRAF+NTRK+MET+RET); period 7: May 27, 2021 and later (EGFR+ALK+ROS1+BRAF+MET+NTRK+RET+KRAS).

For early versus late NGS testing (Figures 10-17), there were greater odds of receiving early NGS-based testing among patients with a later year of initial NSCLC diagnosis, who had no history of smoking, who were in later drug period approval periods, had a PD-L1 test, treated in the MAC J8 WPS, and who had no other biomarker tests or inconclusive testing.

## Discussion

### Overview

This study applied machine learning methods and traditional statistical tools that identified several factors that were significantly associated with not only receiving NGS-based testing but also receiving the testing early when there is a potential for early intervention with targeted therapies. Factors associated with both ever having NGS testing as well as early NGS testing included later year of NSCLC diagnosis, no history of smoking, and evidence of PD-L1 testing. These factors were consistent with the hypothesized direction of candidate variables, as NGS-based testing has been increasing over time, and it was not unexpected that the rate of testing has increased in recent years [4,25]. In addition, consistent with the hypothesized direction of these relationships, patients without a smoking history were more likely to undergo NGS-based testing. The lack of environmental causal factors would lead one to seek other explanations for the onset of lung cancer, including certain genomic abnormalities, which are frequently observed among nonsmokers with lung cancer [26]. PD-L1 testing is generally conducted alongside the NGS test and was only available in later years, so the observation of these relationships was also not unexpected.

### Principal Findings

Factors associated with a greater chance of never receiving NGS testing included older age, lower ECOG performance status, Black race, higher number of single-gene tests, public insurance, and treatment in a geography associated with MolDX Program adoption. Patient age and public insurance are factors that are closely related. Patients aged 65 years and older generally have Medicare coverage, whereas younger patients will have private insurance. The median age of lung cancer diagnosis is 71 years [27], and it is highly likely that a younger patient presenting with NSCLC could raise questions about the genomic aspects of the disease that should be investigated as a result be associated with a higher likelihood of receiving early NGS-based testing as noted in the published literature [28]. Importantly, patient race, similar to prior research [7], remains a significant factor that continues to demonstrate the lack of equity in receipt of NGS-based testing. Of all factors evaluated in this study, racial inequity cannot be explained by any reasonable clinical factors and requires immediate attention by the health care community.

Several factors that did not have a clear association with NGS-based testing were those that also did not have a hypothesized direction associated with a potential relationship. While blood test results may have captured some aspect of well-being, there was no consistent relationship identified. Similarly, while patients with better performance status were more likely to receive NGS-based testing, this relationship was not strong, and the factor was not among those with the highest importance scores observed in this study. Therefore, this study suggests that these factors are likely not largely factored into a decision to receive NGS-based testing and could be why little data were observed in the published literature related to these factors.

The roles of the MolDX program and the MAC region are unclear. The emergence of MAC region J8 WPS as a predictive factor for greater odds of receiving early NGS testing and both JH Novitas and J6 NGS at lower odds of receiving any NGS testing could be an artifact of a large dataset with multiple subgroups or could reflect underlying factors related to this region that could not be explored, given the available data in the electronic data used for this study. Additionally, the timing of MolDX program adoption was not taken into account, so the patients in these regions could have had the decision made at a time that was unrelated to this variable (“yes” or “no”). Other geographic factors such as distance to a clinic, access to testing resources, and site of care could certainly have played a role as well; therefore, the relationship with MolDX should not be overinterpreted. Additionally, not all patients in these regions had Medicare coverage, so there is a great deal of uncertainty in these variables. A study with more comprehensive variables related to patient care in these regions would be needed to come to any clear conclusion about these relationships.

### Limitations

First, this study is based on real-world data. The Flatiron Health deidentified data, as with most other electronic health record–based datasets, do not contain all potentially relevant variables to investigate all aspects of the complex question of NGS-based testing. Factors such as tissue availability, tissue quality, a patient crisis requiring immediate care, and other health care system–related factors were not recorded and may be additional factors that could impact access and receipt of NGS-based testing. The availability of these data, however, would not invalidate the factors that were observed in this study. Second, there were some patients who could have received NGS-based testing at an early stage diagnosis who were not included in this study due to our eligibility criteria, requiring testing within the time frame of advanced or metastatic diagnosis. Therefore, this study may not be generalizable to those diagnosed and tested at earlier stages of the disease. Third, as with all real-world data sources, missingness is a potential issue. However, the rates of NGS-based testing in this study are very similar to other estimates from different data sources, which provides confidence in the outcome variable assessed within the database used for this study [10]. Finally, when evaluating predictive models, a cutoff of 0.5 was applied to the predicted probability of events. While this may result in a suboptimal trade-off of specificity versus sensitivity for certain models (eg, for modeling “early vs late” NGS testing, it resulted in low specificities of ~20% and very high sensitivity of ~98%; Table S2 in Multimedia Appendix 1), the objective of this study was to identify predictors of NGS testing rather than optimizing predictive rules. The probability cutoffs could be further calibrated to strike a desired balance between false positives and negatives.

### Conclusions

Despite the limitations of these data, this study reinforces the need to assure equity in access to NGS-based testing that has been observed in prior research. Black race is consistently associated with lower biomarker testing rates [7]. Other factors may be more associated with disease trajectory (eg, age, lower ECOG performance status, and single-gene tests), emphasizing the flexibility needed in testing for those patients who may not be well enough for systemic therapy or who have an actionable biomarker previously identified. While efforts must be made to ensure all patients diagnosed with NSCLC have equal access to NGS-based testing early in the trajectory of the disease, there may be consideration for the specific patient needs in these cases.

## Data Availability

The data that support the findings of this study have been originated by Flatiron Health, Inc. Requests for data sharing by license or by permission for the specific purpose of replicating results in this manuscript can be submitted to dataaccess@flatiron.com.

## Appendix

Multimedia Appendix 1 Details of study methods, model performance, and variable importance plots.

Multimedia Appendix 2 Computing partial effects from covariates from logistic regression models.

## Abbreviations

ALK: anaplastic lymphoma kinase
BRAF: V-Raf murine sarcoma viral oncogene homolog B
ECOG: Eastern Cooperative Oncology Group
EGFR: epidermal growth factor receptor
ICD: International Classification of Diseases
ICD-9: International Classification of Diseases, Ninth Revision
ICD-10: International Statistical Classification of Diseases, Tenth Revision
KRAS: Kirsten rat sarcoma virus
LASSO: least absolute shrinkage and selection operator
LR: logistic regression
MAC: Medicare Administrative Contractor
MET: mesenchymal epithelial transition
MolDX: Molecular Diagnostics Services
NCCN: National Comprehensive Cancer Network
NGS: next-generation sequencing
NSCLC: non–small cell lung cancer
NTRK: neurotrophic tyrosine receptor kinase
PD-L1: programmed death ligand 1
PLR: penalized logistic regression
RET: rearranged during transfection
ROS1: c-ros oncogene 1
XGBoost: extreme gradient boosting
