# Supplementary material for: Next-Generation Sequencing–Based Testing Among Patients With Advanced or Metastatic Nonsquamous Non–Small Cell Lung Cancer in the United States: Predictive Modeling Using Machine Learning Methods
Source: JMIR Cancer. 2025 Jun 11;11:e64399. doi: 10.2196/64399 (PMC12198702; doi:10.2196/64399)
Supplement: Multimedia Appendix 1 [file cancer_v11i1e64399_app1.docx]

**Table S1.** Hyperparameters of the machine learning models.

| **Models** | **Hyperparameters** |
| --- | --- |
| XGBoost Model | Learning Rate: 0.01  Max Depth: 2:7  Number of Rounds: 10000  Early Stopping Rounds: 200  Minimum Child Weight: 1, 10, 100  Column Subsample Ratio by Tree: 0.5  Number of Features: 146 |
| Logistic Model | Number of Features: 36 |
| LASSO Model | Lambda: iola - Network an optimal penalty parameter (lambda) is selected using grouped lasso penalty (implemented in sparsegl R package)  Number of features: 146 |

**Abbreviations--**LASSO: Least Absolute Shrinkage and Selection Operator; XGBoost: Extreme Gradient Boosting

**Table S2.** Performance of NGS-testing models on validation sets (medians over 1000 splits).

|  | **EVER versus NEVER NGS-tested (N=7538)** | | | | |
| --- | --- | --- | --- | --- | --- |
| **Model Type** | **% AUC**  **(2.5%, 97.5% Percentiles)** | **PR AUC** | **Accuracy** | **Youden Index** | **F1 Score** |
| **XGBoost** | 83.9 (83.1,84.6) | 0.799 | 0.76 | 0.50 | 0.709 |
| **Logistic** | 80.4 (79.5,81.1) | 0.751 | 0.73 | 0.45 | 0.679 |
| **LASSO** | 80.3 (79.4,81.2) | 0.751 | 0.73 | 0.45 | 0.679 |
|  | **NPV** | **PPV** | **Sensitivity** | **Specificity** | **MCC** |
| **XGBoost** | 0.774 | 0.739 | 0.682 | 0.819 | 0.507 |
| **Logistic** | 0.755 | 0.699 | 0.660 | 0.787 | 0.451 |
| **LASSO** | 0.755 | 0.700 | 0.659 | 0.789 | 0.451 |
|  | **EARLY versus LATE NGS-tested (N=3230)** | | | | |
| **Model Type** | **% AUC**  **(2.5%, 97.5% Percentiles)** | **PR AUC** | **Accuracy** | **Youden Index** | **F1 Score** |
| **XGBoost** | 79.5 (77.8,81.2) | 0.948 | 0.86 | 0.19 | 0.920 |
| **Logistic** | 77.7 (75.8,79.4) | 0.942 | 0.85 | 0.17 | 0.917 |
| **LASSO** | 77.2 (73.8, 79.2) | 0.941 | 0.85 | 0.15 | 0.917 |
|  | **NPV** | **PPV** | **Sensitivity** | **Specificity** | **MCC** |
| **XGBoost** | 0.648 | 0.867 | 0.979 | 0.206 | 0.309 |
| **Logistic** | 0.599 | 0.864 | 0.976 | 0.192 | 0.279 |
| **LASSO** | 0.617 | 0.861 | 0.982 | 0.163 | 0.262 |

**Cohort definitions:** *Ever NGS-tested=*Patients in the overall study cohort with evidence of NGS-based biomarker testing in the database; Never NGS-tested=Patients in the overall study cohort with no evidence of NGS-based biomarker testing; *Early NGS-tested*=patients in the *Ever NGS-tested* group whose first or only NGS-based test occurred prior to the start of first-line therapy through Day 7 of first-line therapy; *Late NGS-tested*=patients in the *Ever NGS-tested* group whose first NGS-based test occurred 8 days or later after the start of first-line therapy.

**Abbreviations--%** AUC: Area Under ROC Curve* 100; PR AUC: Area under Precision-Recall curve; LASSO: Least Absolute Shrinkage and Selection Operator; PPV: Positive predictive value; XGBoost: Extreme Gradient Boosting; MCC= Matthew’s correlation coefficient

**Notes:** Accuracy=proportion of correct predictions, Higher AUC=Better distinction between patients EVER versus NEVER or EARLY versus LATE NGS tested; Higher Precision=Better minimization of false positives; Youden Index of >50%, Higher F1 score=Better performance of model; Higher specificity=Better identification of negative results

**Table S3.** Performance of Final Logistic Regression NGS-testing models on testing set.

| **Model Outcome** | **% AUC** | **R-squared** | **Brier Score^*^** | **AIC** | **BIC** |
| --- | --- | --- | --- | --- | --- |
| **Ever versus Never NGS-tested** | 80.0 | 0.208 | 0.179 | 6883 | 7490 |
| **Early versus Late NGS-tested** | 81.0 | 0.110 | 0.107 | 1937 | 2237 |

**Cohort definitions:** *Ever NGS-tested=*Patients in the overall study cohort with evidence of NGS-based biomarker testing in the database; Never NGS-tested=Patients in the overall study cohort with no evidence of NGS-based biomarker testing; *Early NGS-tested*=patients in the *Ever NGS-tested* group whose first or only NGS-based test occurred prior to the start of first-line therapy through Day 7 of first-line therapy; *Late NGS-tested*=patients in the *Ever NGS-tested* group whose first NGS-based test occurred 8 days or later after the start of first-line therapy.

**Abbreviations**: AUC=Area Under Curve; AIC=Akaike information criterion; BIC=Bayesian information criterion

**Notes:** Higher AUC=Better distinction between patients Never versus Ever or Early versus Late NGS tested; Higher R-squared=better model fit; Lower AIC/BIC=Better model fit of false positives

**Figure S1.** Variable* importance plots for EVER versus NEVER NGS-tested:

LASSO top 24 variables: median co-efficient (with 95% replication interval (RI)) on 20% validation data from 1000 random splits.


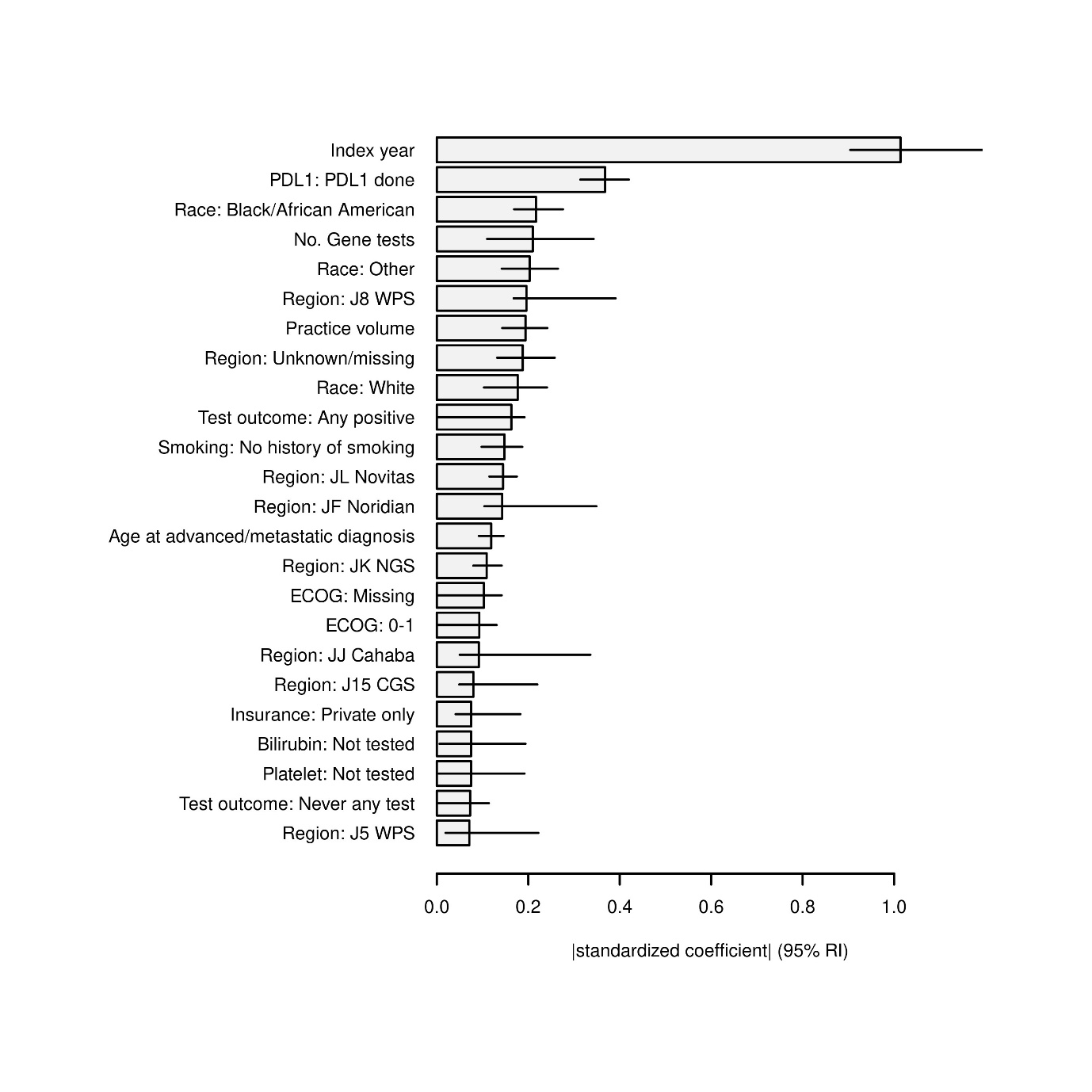


**Cohort definitions:** *Ever NGS-tested=*Patients in the overall study cohort with evidence of NGS-based biomarker testing in the database; Never NGS-tested=Patients in the overall study cohort with no evidence of NGS-based biomarker testing; *Early NGS-tested*=patients in the *Ever NGS-tested* group whose first or only NGS-based test occurred prior to the start of first-line therapy through Day 7 of first-line therapy; *Late NGS-tested*=patients in the *Ever NGS-tested* group whose first NGS-based test occurred 8 days or later after the start of first-line therapy.

*May include only some levels of the covariates.

**Figure S2.** Variable* importance plots for EARLY versus LATE NGS-tested: LASSO top 24 variables: Median co-efficient (with 95% replication interval (RI)) on 20% validation data from 1000 random splits.


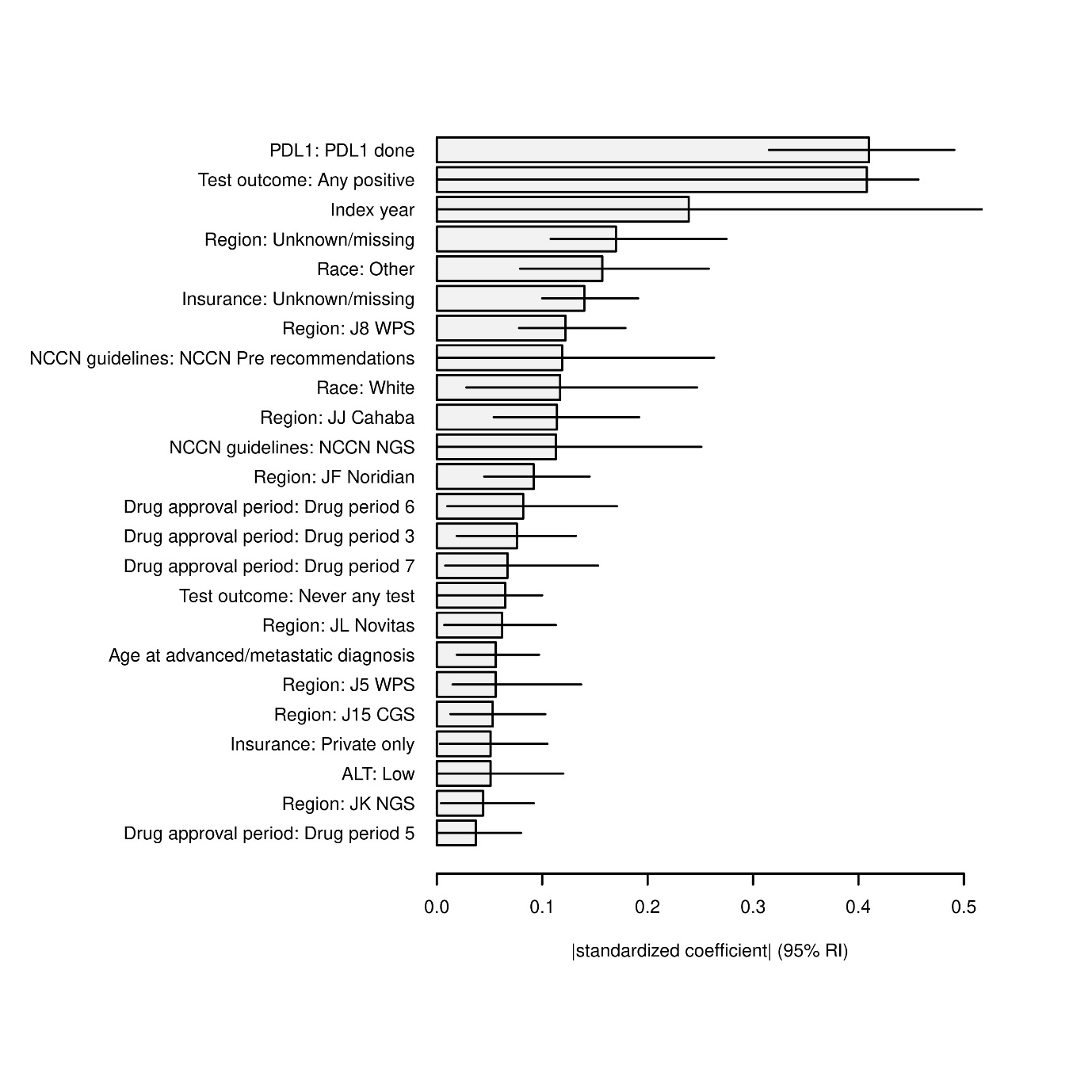


**Cohort definitions:** *Ever NGS-tested=*Patients in the overall study cohort with evidence of NGS-based biomarker testing in the database; Never NGS-tested=Patients in the overall study cohort with no evidence of NGS-based biomarker testing; *Early NGS-tested*=patients in the *Ever NGS-tested* group whose first or only NGS-based test occurred prior to the start of first-line therapy through Day 7 of first-line therapy; *Late NGS-tested*=patients in the *Ever NGS-tested* group whose first NGS-based test occurred 8 days or later after the start of first-line therapy.

*May include only some levels of the covariates.
