# Supplementary material for: Next-Generation Sequencing–Based Testing Among Patients With Advanced or Metastatic Nonsquamous Non–Small Cell Lung Cancer in the United States: Predictive Modeling Using Machine Learning Methods
Source: JMIR Cancer. 2025 Jun 11;11:e64399. doi: 10.2196/64399 (PMC12198702; doi:10.2196/64399)
Supplement: Multimedia Appendix 2 [file cancer_v11i1e64399_app2.docx]

In applying machine learning models to data it is often of interest to compute partial effects of continuous or categorical covariates while averaging over all other potential confounders. Assuming no unmeasured confounder such effects have causal interpretation (See Zhao, Q. and T. Hastie (2021). "Causal interpretations of black-box models." Journal of Business & Economic Statistics **39**(1): 272-281). For example, in a regression model with $p$ covariates $X_{1},\ldots,X_{p}$ and their interactions we may be interested in computing a partial effect of a categorical variable $X_{k}$fixed at level $j$versus a reference level $J$ while averaging all other covariates at their actual observed values in the data set. To address causal effects we would need to compute expected potential (hypothetical) outcomes for the $i$ ^th^ patient $\hat{Y}_{i}^{\left( X_{ik}=j,\boldsymbol{X}_{i}^{\left( -k \right)}=\boldsymbol{x}_{i,obs}^{\left( -k \right)} \right)}$ by first fixing covariate $X_{k}$ at $j$^th^ level while letting all the $p-1$ remaining covariates $\boldsymbol{X}^{\boldsymbol{(-}k\boldsymbol{)}}$ to assume the actual values for all N subjects in training data; then similarly fixing by first fixing covariate $X_{k}$ at $J$^th^ level and computing the difference

$\hat{\delta}_{jJ}^{k}=\frac{1}{N}\sum_{i=1}^{N} \left( Y_{i}^{(X_{ik}=j,\boldsymbol{X}_{i}^{(-k)}=\boldsymbol{x}_{i,obs}^{(-k)})}-Y_{i}^{(X_{ik}=J,\boldsymbol{X}_{i}^{(-k)}=\boldsymbol{x}_{i,obs}^{(-k)})} \right)$.

To be specific, consider now a logistic regression with two categorical variables and their interactions. Let $X_{1}\in\{1,\ldots, J\}$ be represented by $J-1$ binary (dummy) variables $X_{11,}\ldots, X_{1,J-1}\in\left\{ 0,1 \right\}$with 𝐽 as the reference level. Similarly let $X_{2}\in\{1,\ldots, K\}$ be represented by $K-1$binary (dummy) variables $X_{21,}\ldots, X_{2,K-1}\in\left\{ 0,1 \right\}$with $K$ as the reference level. The logistic regression with main effects of $X_{1}$ and $X_{2}$and interaction terms is represented as

$log(p/(1-p))=a_{0}+\sum_{j=1}^{J-1} a_{1j}X_{1j}+\sum_{k=1}^{K-1} a_{2k}X_{2k}+\sum_{j=1}^{J-1} \sum_{k=1}^{K-1} a_{3jk}X_{1j}X_{2k}$.

The log odds for levels 1$\leq j<J$ vs. reference level $J$ of variable $X_{1}$expressed as the contrast in estimated average potential outcomes:

$\hat{\delta}_{j}=\hat{a}_{1j} +\sum_{k=1}^{K-1} \hat{a}_{3,j,k}f_{2,k}$,

where $f_{2,k}=n^{-1}\sum_{i=1}^{n} I(X_{2}=k)$ is the proportion of $X_{2}$ with category “$k$” in training data and $\hat{a}$’s designate the maximum likelihood estimates of the coefficients in the above logistic regression model.

Let ${\hat{\boldsymbol{b}}}_{j}=\left( \hat{a}_{1j}, \hat{a}_{3j1},...,\hat{a}_{3jK-1} \right), j=1,..,J-1$ is a *k*-dimensional vector for $j$^th^ contrast, and $\boldsymbol{w}=(1,f_{2,1},..,f_{2,K-1})$. Let, $\boldsymbol{v}_{j}$ be a $K\times K$matrix of covariances $\boldsymbol{v}_{j}=cov\left( \hat{b}_{jk},\hat{b}_{jl} \right), k,l=1,..,K$. Using this notation, the target estimator $\hat{\delta}_{j}$and its standard error can be expressed as $\hat{\delta}_{j}={\hat{\boldsymbol{b}}}_{j}^{T}\boldsymbol{w}$, ${se(\hat{\delta}}_{j})={{(\boldsymbol{w}^{T}\boldsymbol{v}}_{j}\boldsymbol{w)}}^{1/2}$**.**

Similarly, we can derive partial effects and their standard errors for other more complex scenarios for models with continuous and categorical variables and their interactions. We used standard SAS proc logistic to obtain estimates of coefficients and their standard errors and a custom code in SAS proc iml to carry out computation of $\hat{\delta}_{j}$ and associated standard errors.
